# NF-κB-mediated developmental delay extends lifespan in *Drosophila*

**DOI:** 10.1073/pnas.2420811122

**Published:** 2025-05-08

**Authors:** Ping Kang, Peiduo Liu, Yanhui Hu, Jinoh Kim, Ankur Kumar, Marlene K. Dorneich-Hayes, Wren Murzyn, Zenessa J. Anderson, Lexi N. Frank, Nicholas Kavlock, Elizabeth Hoffman, Chad C. Martin, Ting Miao, MaryJane Shimell, Jo Anne Powell-Coffman, Michael B. O’Connor, Norbert Perrimon, Hua Bai

**Affiliations:** ^a^Department of Genetics, Development, and Cell Biology, Iowa State University, Ames, IA 50011; ^b^Department of Genetics, Blavatnik Institute, Harvard Medical School, Harvard University, Boston, MA 55455; ^c^Department of Genetics, Cell Biology and Development, University of Minnesota, Minneapolis, MN 02115; ^d^HHMI, Boston, MA 02115

**Keywords:** developmental theory of aging, PTTH, NF-κB, innate immunity, oenocyte

## Abstract

Developmental time (or time to maturity) strongly correlates with an animal’s maximum lifespan, with late-maturing individuals often living longer. However, the genetic mechanisms underlying this phenomenon remain largely unknown. This may be because most previously identified longevity genes regulate growth rate rather than developmental time. To address this gap, we genetically manipulated prothoracicotropic hormone (PTTH), the primary regulator of developmental timing in *Drosophila*, to explore the genetic link between developmental time and longevity. Loss of *PTTH* delays developmental timing without altering the growth rate. Intriguingly, *PTTH* mutants exhibit extended lifespan despite their larger body size. This lifespan extension depends on ecdysone signaling, as feeding 20-hydroxyecdysone to *PTTH* mutants reverses the effect. Mechanistically, loss of *PTTH* blunts age-dependent chronic inflammation, specifically in fly hepatocytes (oenocytes). Developmental transcriptomics reveal that NF-κB signaling activates during larva-to-adult transition, with PTTH inducing this signaling via ecdysone. Notably, time-restricted and oenocyte-specific silencing of *Relish* (an NF-κB homolog) at early 3rd instar larval stages significantly prolongs adult lifespan while delaying pupariation. Our study establishes an aging model that uncouples developmental time from growth rate, highlighting NF-κB signaling as a key developmental program in linking developmental time to adult lifespan.

## Significance

Despite the strong link between animal development and adult lifespan, how developmental programs influence longevity remains poorly understood. Here, we show that mutants of the insect neuropeptide hormone prothoracicotropic hormone (PTTH) exhibit delayed developmental timing and extended adult lifespan. Intriguingly, PTTH loss reduces innate immune signaling during *Drosophila* development via the steroid hormone ecdysone. Moreover, temporal and spatial inactivation of developmental innate immune signaling extends both developmental time and lifespan. Our findings reveal a neuropeptide–steroid hormone–immunity axis in lifespan control, offering valuable insights into the role of developmental programs in regulating adult longevity.

Aging is commonly viewed as a progressive deterioration of physiological function and accumulation of stochastic damage with age. However, mounting evidence suggests that developmental processes also influence aging outcomes. For example, developmental timing and growth rate are known to be associated with adult lifespan (1, 2). Genetic perturbation of animal development and growth programs, such as growth hormone (GH) in mammals or insulin/insulin-like growth factor signaling (IIS) in invertebrates, often lead to retarded growth and prolonged adult lifespan (3–5). In addition, a number of previous lifespan studies in model organisms further support the existence of developmental programs as determinants of adult lifespan. For example, early-age treatment of GH reverses the lifespan extension of Ames dwarf mice (GH deficiency) (6). In addition, the constitutive knockdown of complex I subunit NDUFS1/ND75 in muscles led to reduced systemic insulin signaling and extended lifespan in *Drosophila* (7). Further, transient exposure to low dosages of oxidants during development extends the adult lifespan of *Drosophila* and *Caenorhabditis elegans* (8, 9).

Final body size at developmental maturity often correlates with maximum lifespan (10–13). For instance, smaller individuals within a species often live longer than larger ones (11). In contrast, the species with larger body sizes live longer than those with smaller body sizes (12). However, there are some exceptions; for example, bats are extremely long-lived for their size, up to 30 y of age (13). This might be due to their slow rate of development and growth. Depending on the species, it could take 6 to 24 mo for bats to reach sexual maturity, whereas mice reach sexual maturity at 6 to 8 wk of age and have a maximum lifespan of 2 to 3 y (14, 15). These observations suggest that developmental time (time to maturity), rather than body size alone, may drive lifespan differences. Body size reflects both growth rate and developmental duration, yet comparative studies show a robust positive correlation between developmental time and maximum lifespan (1, 14), while postnatal growth rate shows only moderate correlation in mammals and none in birds (1). Existing genetic aging models, targeting growth regulators like GH, insulin/IGF-1, and mechanistic target of rapamycin complex 1 (mTORC1) (3, 5, 16), typically alter both growth rate and developmental time, obscuring their individual contributions to lifespan extension. Resolving this requires a model that isolates developmental timing from growth rate.

In the model organism *Drosophila melanogaster*, the developmental timing is controlled by prothoracicotropic hormone (PTTH). In *Drosophila*, tissue growth and body size are achieved through multiple larval molts. At the end of larval development, animals transform into sexually mature adults through a unique process called metamorphosis (17–19). Insect molting and metamorphosis are orchestrated by the steroid hormone ecdysone, which is synthesized and released from the prothoracic gland (PG) (20). PTTH is the major driver of ecdysone biosynthesis. PTTH is secreted by a few neuroendocrine cells in each brain hemisphere and signals through the receptor tyrosine kinase Torso to activate mitogen-activated protein (MAP) kinase signaling within the PG (21, 22). PTTH belongs to the cystine knot family of growth factors (23). Although PTTH has no clear mammalian ortholog, it has been proposed that PTTH may play similar roles as mammalian gonadotropin-releasing hormone (GnRH) in controlling the timing of sexual maturity (24, 25). Loss of *Ptth* results in slower kinetics of ecdysone production, a delay in developmental timing, and slow imaginal disc growth (21, 26). Interestingly, loss of *Ptth* does not affect animal growth rate. As a result, *Ptth* mutants are significantly larger than those of wild-type flies (21, 26). Unlike genetic models that conflate growth rate and timing, *Ptth* mutants offer a unique system to probe the specific link between developmental time and adult lifespan, free from growth rate confounds.

Age-associated chronic inflammation, also known as inflammaging, is one of the major hallmarks of aging (27, 28). Inflammatory cytokines, such as interleukin 6 (IL-6) and tumor necrosis factor α (TNF-α), are often induced during aging, and elevated IL-6 in the circulation is a powerful indicator of all-cause mortality in aging human populations (29, 30). Chronic inflammation is not only a biomarker of aging, but also drives aging and age-related pathologies. Anti-inflammatory interventions often preserve tissue function and slow aging processes. For example, inhibition of TNF-α signaling rescues premature aging phenotypes in mice with Tfam-deficient T cells (31). Further, brain-specific knockout of *IKKβ* in mice (32) or glial-specific knockdown of *Relish/NF-κB* in *Drosophila* prolongs lifespan (33). Recently, genetic or pharmacologic inhibition of proinflammatory cytokine IL-11 significantly extends the life expectancy of male and female mice (34).

Besides the extensive studies of NF-κB in inflammation at the adult stage, however, the role of NF-κB signaling in animal development is largely unexplored. Recently, Relish/NF-κB has been found to be expressed in the hematopoietic niche during larval development in *Drosophila*, and to play a vital role in maintaining the blood progenitors in developing lymph glands (35). Intriguingly, silencing NF-κB signaling specifically at the *Drosophila* pupal stage enhances the susceptibility of adult flies to viral infection, indicating that NF-κB signaling during metamorphosis is essential in conditioning adult antiviral responses (36).

In this study, we utilized loss-of-function mutants of *Drosophila Ptth* as a model to dissect the genetic mechanisms underlying the link between developmental timing and lifespan regulation. We surprisingly find that *Drosophila Ptth* mutants, which exhibit a longer developmental time but normal growth rate, are long-lived and stress tolerant. The lifespan extension of *Ptth* mutants is dependent on age-dependent inactivation of NF-κB in fly hepatocytes (oenocytes). Intriguingly, we found that NF-κB signaling is activated at early pupal stages, and which is attenuated in *Ptth* mutants. Intriguingly, oenocyte- and pupal-specific knockdown of NF-κB significantly extends lifespan. Taken together, our findings uncover a genetic mechanism linking developmental time (time to maturity) to adult lifespan, as well as an unexpected role of developmental NF-κB signaling in shaping adult physiology.

## Results

### Loss of *Ptth* Prolongs Lifespan and Healthspan in *Drosophila*.

**Fig. 1.**
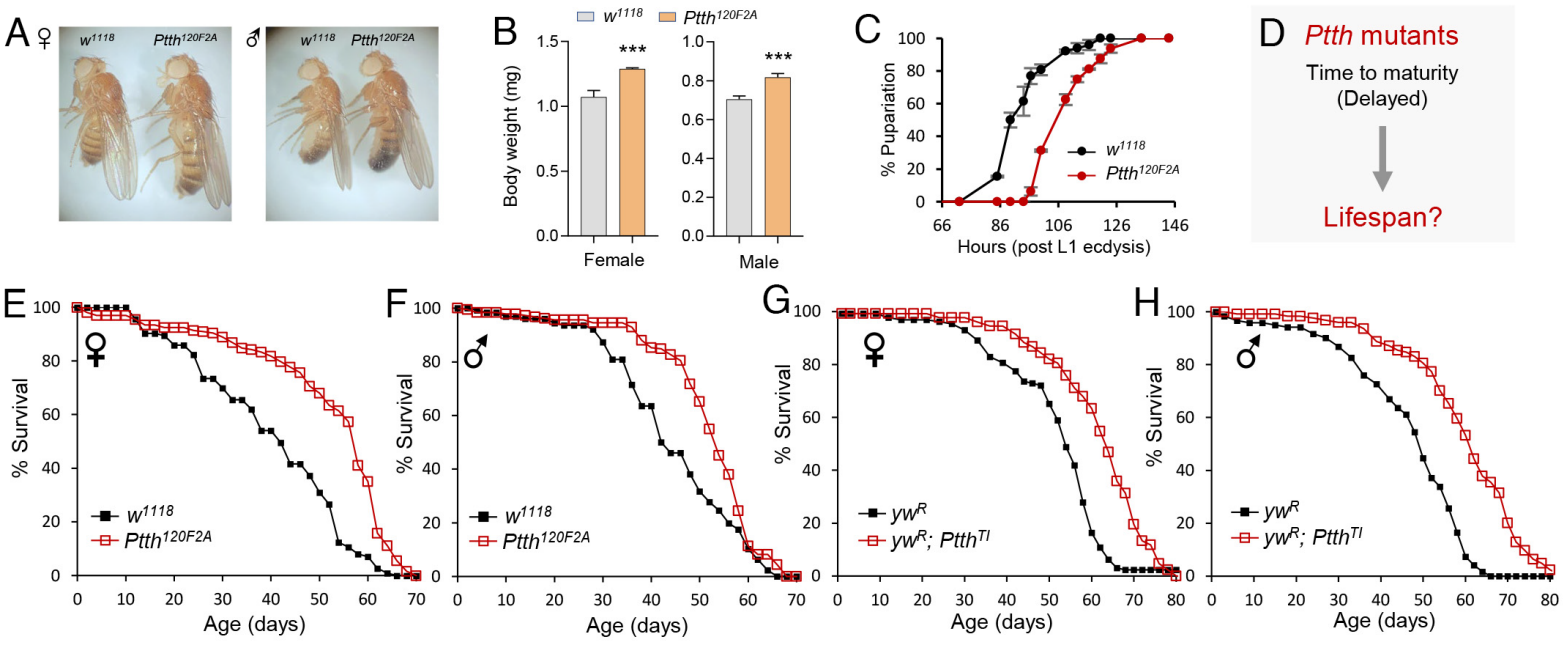
Loss of *Ptth* prolongs lifespan in *Drosophila*. (*A*) Stereomicroscopy images showing larger body size of *Ptth* mutants (*Ptth^120F2A^*). (*B*) Body weight of 1-wk-old adult wild-type (*w^1118^*) and *Ptth* mutants (*Ptth^120F2A^*). Unpaired *t* test, ***P < 0.001, n = 4 (20 adults per replicate). (*C*) Developmental timing analysis of wild-type (*w^1118^*) and *Ptth* mutants (*Ptth^120F2A^*). Three replicates were performed for each genotype (about 30 to 40 larvae each replicate). (*D*) Research question to be investigated in this study. (*E* and *F*) Lifespan analysis of the null alleles *Ptth^120F2A^*. Log-rank test (vs. *w^1118^*). Female: *P* < 0.001, n = 197. Male: *P* < 0.001, n = 184. (*G* and *H*) Lifespan analysis of the null allele *Ptth^TI^*. Log-rank test (vs. *yw^R^*). Female: *P <* 0.05, n = 128. Male: *P* < 0.001, n = 123.

To genetically determine the role of developmental time in longevity regulation, we utilized two previously generated loss-of-function alleles of *Ptth*, the key hormonal factor that regulates developmental timing in *Drosophila* (26, 37). *Ptth^120F2A^*, a null allele with seven bp deletion in the final exon of *Ptth*, was previously generated through TALEN-directed mutagenesis (26) (*SI Appendix*, Fig. S1*A*). *Ptth^TI^*, another null allele with all the exons replaced by a 3P3-RFP cassette through CRISPR-Cas9 and homologous recombination-mediated gene targeting (37) (*SI Appendix*, Fig. S1*A*). Before the lifespan analysis, both *Ptth* alleles were backcrossed to matched wild-type background for five generations to eliminate the confounding effects of genetic background. Consistent with previous studies, *Ptth* mutants exhibited larger body size (Fig. 1*A*) and increased body weight in both sexes (Fig. 1*B* and *SI Appendix*, Fig. S1*B*). This was because the loss of Ptth resulted in delayed developmental timing (time to pupariation), about 20 h of delay (Fig. 1*C* and *SI Appendix*, Fig. S1*C*), but no change in growth rate (26).

Utilizing the *Ptth* alleles that specifically disrupted developmental time, but not growth rate, we then asked whether loss of *Ptth* alters adult lifespan (Fig. 1*D*). Notably, the *Ptth* mutants, which exhibited larger body size and delayed developmental time, were long-lived compared to their matched controls (both females and males, 20 to 38% extension of median lifespan, Fig. 1 *E*–*H*), and showed reduced age-specific mortality (*SI Appendix*, Fig. S1*D*). Consistent with the extended lifespan, *Ptth* mutants showed increased resistance to paraquat-induced oxidative stress (*SI Appendix*, Fig. S1 *E* and *F*), and preserved climbing ability during aging (*SI Appendix*, Fig. S1 *G* and *H*). Importantly, *Ptth* mutants prolong their lifespan without any reproductive cost. In fact, the female fecundity of *Ptth* mutants was higher than that of wild-type flies (*SI Appendix*, Fig. S2*A*).

The observed large body size and increased fecundity in long-lived *Ptth* mutants seemingly contradicts most of the longevity mutants in previous literature. However, it is not entirely unexpected. In one of our previous studies, we showed that heterozygous mutants of *chico*, the fly homolog of insulin receptor substrate 1 (IRS1), were long-lived, despite increased fecundity (38). Despite potential increased metabolic demands, longevity persists, possibly due to efficient resource allocation. To further characterize the unusual *Ptth* mutants, we measured the feeding behavior, L-amino acid and TCA cycle intermediates, and locomotor activity. Interestingly, *Ptth* mutants did not show reduced food intake, rather they ate more food than wild-type flies (*SI Appendix*, Fig. S2*B*). In addition, *Ptth* mutants had elevated L-amino acids, but the major TCA cycle intermediates (citrate and malate) remained unchanged (*SI Appendix*, Fig. S2*C*). We also did not observe a significant change in locomotor activity between *Ptth* mutants and wild-type flies (*SI Appendix*, Fig. S2*D*). Thus, we established *Ptth* mutants as an aging model to dissect the mechanisms linking developmental time to lifespan regulation. The long-lived *Ptth* mutants exhibit many unexpected characteristics compared with previous longevity mutants.

### *Ptth* Mutants Repress Age-Dependent Upregulation of Innate Immune Signaling.

To understand the molecular mechanisms underlying PTTH-regulated longevity and how delayed developmental time prolongs adult lifespan, we performed a bulk RNA-Seq analysis to characterize the transcriptomic changes in young (5-d-old) and aged (38-d-old) female wild-type (*w^1118^*) and *Ptth* mutants (*Ptth^120F2A^*). We identified 731 differentially expressed genes (DEGs) between *Ptth* mutants and wild-type at young age (fold change > 1.5, FDR < 0.05) (*SI Appendix*, Fig. S1*I*). Among them, 129 were significantly upregulated, while 602 were significantly downregulated. Gene ontology (GO) analysis showed that these genes were enriched in biological processes including digestive system development, mesoderm development, epithelial tube morphogenesis, and tissue morphogenesis and development (*SI Appendix*, Fig. S1*J*). On the other hand, there were 610 DEGs between *Ptth* mutants and wild-type at old age (fold change > 1.5, FDR < 0.05) (*SI Appendix*, Fig. S1*K*). Surprisingly, almost all the enriched pathways identified from the aged flies were related to innate immunity (*SI Appendix*, Fig. S1*L*).

**Fig. 2.**
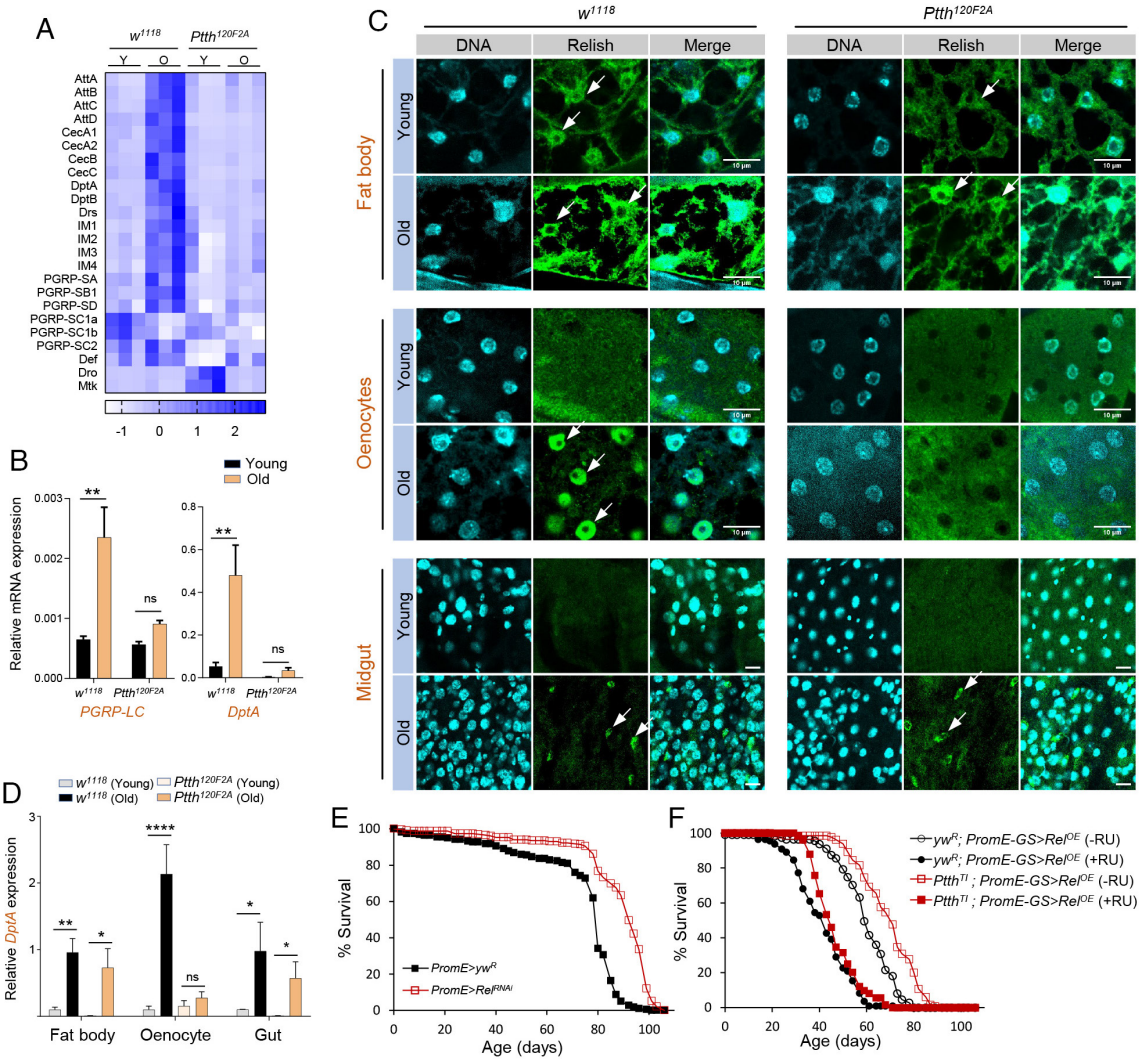
Loss of *Ptth* blocks age-dependent activation of NF-κB signaling specifically in fly oenocytes. (*A*) Heat map showing genes in innate immunity pathway are differentially regulated by *Ptth* mutants with age. Y: young age; O: old age. (*B*) qPCR analysis of the expression of *PGRP-LC* and antimicrobial peptide Diptericin A (*DptA*) in young and old *Ptth* mutants and wild-type (*w^1118^*). Two-way ANOVA followed by Bonferroni’s multiple comparison test. ns, not significant; **P < 0.01, n = 3. (*C*) Immunostaining analysis of nuclear translocation of Relish/NF-κB in young and old *Ptth* mutants and wild-type (*w^1118^*). Three tissues were analyzed, oenocytes, midgut, fat body. (Scale bar: 10 μm.) White arrow: nuclear localized Relish. (*D*) qPCR analysis of the expression of *DptA* in three different fly tissues dissected from young and old *Ptth* mutants and wild-type (*w^1118^*). Two-way ANOVA followed by Bonferroni’s multiple comparison test. ns, not significant; *P < 0.05; **P < 0.01; ****P < 0.0001, n = 3 ~ 6. (*E*) Oenocyte-specific knockdown of *Relish/NF-κB* extends lifespan (female). Log-rank test, *P* < 0.001, total n = 452. (*F*) Oenocyte-specific overexpression of *Relish/NF-κB* blocked the lifespan extension of *Ptth* mutants (female). Log-Rank test, *P* < 0.001, total n = 475.

To gain insights into the biological processes induced in aged wild-type flies but not in the *Ptth* mutants, we analyzed the age-associated DEGs in both wild-type and *Ptth* mutants, respectively. Among the 1,220 age-associated DEGs found in wild-type flies (fold change > 2, FDR < 0.05), 754 of them were differentially expressed only in aged wild-type flies, but not in aged *Ptth* mutants (244 upregulated and 510 downregulated) (*SI Appendix*, Fig. S1*M*). GO analysis revealed that the 244 age-upregulated DEGs found only in wild-type were again enriched for immune response and defense to bacterium (*SI Appendix*, Fig. S1*M*). Aging induced most of the antimicrobial peptides (AMPs) (e.g., *AttA, AttB, AttC, CecA1, CecA2, CecB, CecC, DptA, DptB, Drs*) and immune peptide Bomanin (e.g., *IM1/BomS1, IM2/BomS2, IM3/BomS3, IM4/Dso1*) in wild-type flies, but not in *Ptth* mutants (Fig. 2*A*). Thus, loss of *Ptth* blocks the age-dependent induction of both Imd and Toll innate immunity pathways. Further, we verified these findings using qRT-PCR. The expression of both peptidoglycanrecognition protein LC (*PGRP-LC*) and *DptA*, two major players of the Imd pathway, was significantly upregulated upon normal aging in wild-type flies, while loss of *Ptth* alleviated these age-related inductions (Fig. 2*B*). As the hyperactivation of innate immune pathways is a hallmark of chronic inflammation (inflammaging), it suggests that slow developing *Ptth* mutants prolong lifespan by suppressing inflammaging.

To test whether decreased innate immunity in *Ptth* mutants could result in any cost of animal fitness, in particular in fighting bacterial infections, we challenged young wild-type and *Ptth* mutants with the Gram-negative pathogenic bacterium *Erwinia carotovora carotovora 15* (*Ecc15*). Surprisingly, *Ptth* mutants were more tolerant to *Ecc15* infection than wild-type flies (*SI Appendix*, Fig. S3*A*). *Ecc15* challenges upregulated innate immunity in both wild-type and *Ptth* mutants, as indicated by elevated transcription of *PGRP-LC* and *DptA* (*SI Appendix*, Fig. S3*B*). Intriguingly, the degree of such induction was much lower in *Ptth* mutants (*SI Appendix*, Fig. S3*B*). In addition, we also examined the inflammatory signaling, such as JAK-STAT signaling, in *Ptth* mutants upon *Ecc15* challenges. Interestingly, *Ptth* mutants significantly attenuated *Ecc15*-induced JAK-STAT signaling, as indicated by the messenger RNA (mRNA) expression of *upd3* (the homolog of mammalian IL-6) and *Socs36E* (*SI Appendix*, Fig. S3*C*). JAK-STAT signaling is known to be activated in response to gut epithelial cell damage during bacterial infection and plays an essential role in intestinal repair (39). The low levels of JAK-STAT activation upon *Ecc15* challenge indicates that loss of *Ptth* protects gut epithelial cells from damage through optimal levels of immune response and reduced chronic inflammation during bacterial infection. The reduced chronic inflammation, indicated by the expression of *upd3/IL-6* and *Socs36E*, was also found in aged *Ptth* mutants when compared to wild-type flies (*SI Appendix*, Fig. S3*D*). Together, these results demonstrate that *Ptth* mutants exhibit robust immune defense capacity with well-balanced innate immunity activation and reduced chronic inflammation during bacterial infection and aging.

### PTTH Regulates Relish/NF-κB Signaling Specifically in Fly Hepatocytes.

PTTH is a neuropeptide hormone secreted by two bilateral small populations of neuroendocrine cells (PG neurons) in the larval brain. It can act as a neurotransmitter that is transported from PG neurons to PG to promote ecdysone biosynthesis (21). On the other hand, it behaves as a systemic hormone that travels through the circulation to target distal tissues (e.g., light-sensing organs) to modulate larval light avoidance behavior (40). To further understand the mechanisms underlying PTTH-regulated longevity, we decided to identify the target tissues of PTTH.

We first measured the age-dependent induction of *DptA* in various fly body parts. We found that among three body parts (heads, thorax, and abdomen), loss of *Ptth* only blocked age-dependent induction of *DptA* in the fly abdomen (*SI Appendix*, Fig. S4*A*). Next, we examined Relish/NF-κB activation (indicated by Relish nuclear translocation) (41, 42) and *DptA* mRNA expression in three major abdominal tissues of adult flies, fat body, oenocytes, and gut. Relish is known to be cleaved upon innate immune activation, and the 68 kDa N-terminal fragment (Rel68) is translocated to the nucleus to activate the transcription of AMP genes (e.g., *DptA*) (41). Using an antibody specifically recognizing Rel68, we found that during aging the nuclear translocation of Relish was enhanced in all three tissues (Fig. 2*C*). Interestingly, loss of *Ptth* attenuated age-dependent induction of Relish nuclear translocation only in oenocytes (Fig. 2*C*). Consistently, we found that *Ptth* mutants blocked the age-related induction of *DptA* expression in oenocytes, but not fat body and gut (Fig. 2*D*). Further, age-dependent activation of JAK-STAT signaling, as indicated by the expression of *upd3/IL-6* and *Socs36E*, was also blocked by *Ptth* mutants specifically in oenocytes (*SI Appendix*, Fig. S4 *B* and *C*). Taken together, these findings suggest that PTTH regulates age-dependent activation of innate immunity and inflammatory signaling specifically in fly oenocytes, the homolog of mammalian hepatocytes.

### Hepatic Relish/NF-κB Is Required for the Lifespan Extension of *Ptth* Mutants.

NF-κB signaling has been shown to regulate longevity through the central nervous system. Brain-specific knockout of *IKKβ* in mice (32) or glial-specific knockdown of *Relish/NF-κB* in flies (33) prolongs lifespan. However, it remains to be determined whether hepatic NF-κB signaling also contributes to longevity. Thus, we knocked down *Relish* specifically in oenocytes using the oenocyte-specific GAL4 driver (*PromE-GAL4*). Strikingly, constitutive oenocyte-specific knockdown of *Relish* extended the fly lifespan (Fig. 2*E*).

**Fig. 3.**
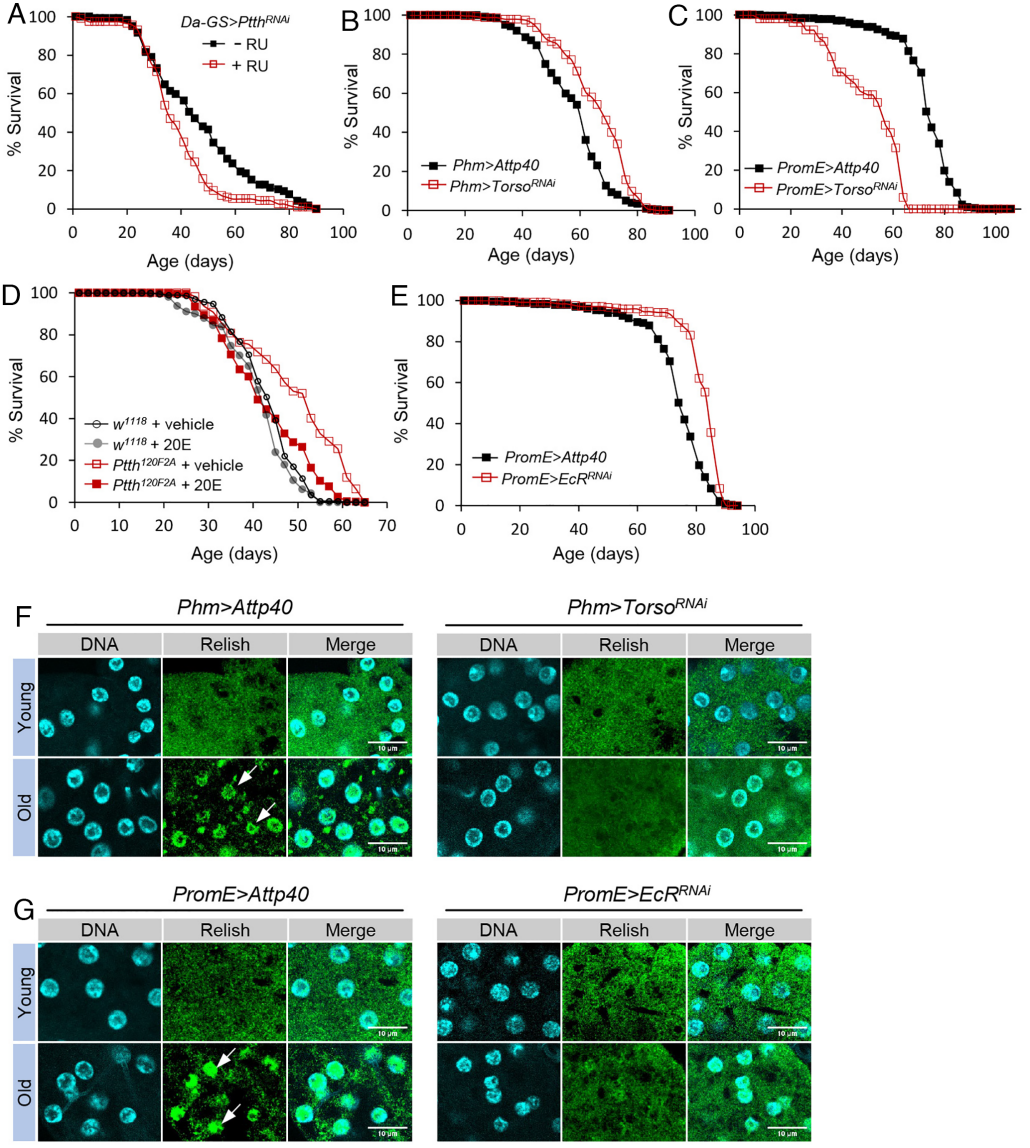
Silencing PTTH receptor *Torso* and ecdysone receptor prolongs lifespan. (*A*) Lifespan analysis of adult-onset knockdown of *Ptth* (female). Log-rank test, *P* < 0.001, n = 234. RU486 (mifepristone, or RU) was used to activate Da-GS-GAL4 GeneSwitch driver. (*B*) PG-specific knockdown of PTTH receptor *Torso* extends lifespan (female). Log-rank test, *P* < 0.001, n = 475. (*C*) Oenocyte-specific knockdown of PTTH receptor *Torso* shortens lifespan (female). Log-rank test, *P* < 0.001, n = 280. (*D*) 20E feeding during 3rd instar larval stage rescued lifespan extension phenotype of *Ptth* mutants (female). Log-rank test (*Ptth^120F2A^* vs. other groups), *P* < 0.001, n = 554. (*E*) Oenocyte-specific knockdown of ecdysone receptor (*EcR*) extends lifespan (female). Log-rank test, *P* < 0.001, n = 471. (*F*) Immunostaining analysis of nuclear translocation of Relish/NF-κB in oenocytes of young and old control (*Phm>Attp40*) and PG-specific *Torso* knockdown flies (*Phm>Torso^RNAi^*). (Scale bar: 10 μm.) White arrow: nuclear localized Relish. (*G*) Immunostaining analysis of nuclear translocation of Relish/NF-κB in oenocytes of young and old control (*PromE>Attp40*) and PG-specific *Torso* knockdown flies (*PromE>EcR^RNAi^*). (Scale bar: 10 μm.) White arrow: nuclear localized Relish.

Given that PTTH regulates age-dependent nuclear translocation of Relish in oenocytes, we tested whether the lifespan extension of *Ptth* mutants is dependent on oenocyte-specific Relish/NF-κB signaling. We combined *Ptth* mutants with the oenocyte-specific GeneSwitch GAL4 driver (*PromE-GS-GAL4*), then crossed it with a *UAS-FLAG-Rel68* line that carries a constitutively active form of Relish. Ectopic expression of *Relish* shortened the lifespan of both groups, but oenocyte-specific overexpression of *Relish* completely abolished the lifespan extension in *Ptth* mutants (Fig. 3*D*), suggesting a specific genetic interaction between PTTH and Relish in lifespan regulation (Fig. 2*F*). Together, our data suggest that PTTH regulates lifespan through oenocyte-specific Relish/NF-κB signaling.

### Silencing PTTH Receptor *Tors*o and Ecdysone Receptor Prolongs Lifespan.

*Ptth* expresses throughout the last instar larval, pupal, and adult stages. But its expression rises about 12 h before pupariation and peaks at prepupal and early pupal stages (*SI Appendix*, Fig. S5*A*) (21). To determine whether PTTH acts during adulthood or during development to regulate longevity, we performed a lifespan analysis on flies with adult-onset knockdown of *Ptth*. A ubiquitous GeneSwitch driver (*Da-GS-GAL4*) was used to drive adult-onset and global knockdown of *Ptth*. Interestingly, adult-specific knockdown of *Ptth* shortened the fly lifespan (Fig. 3*A*), which suggests that PTTH may regulate lifespan during development, rather than during adulthood. RU486 feeding alone did not alter the fly lifespan (*SI Appendix*, Fig. S4*D*).

During larval and early pupal stages, the receptor tyrosine kinase Torso mediates PTTH signaling by activating a MAP kinase cascade within the PG to initiate ecdysone biosynthesis and metamorphosis (the juvenile-to-adult transition) (22, 26). The PG degenerates during pupal development and is completely lost at the onset of adulthood (43). We then asked whether PTTH regulates lifespan through its receptor Torso in the PG during *Drosophila* development. Using a PG-specific GAL4 driver (*Phm-GAL4*), we found that PG-specific knockdown of *Torso* prolonged the fly lifespan (Fig. 3*B*). Further, PG-specific knockdown of *Torso* blocked age-dependent increases in nuclear translocation of Relish in oenocytes (Fig. 3*F*). We then asked whether PTTH can signal directly through Torso within oenocytes to regulate lifespan. Interestingly, oenocyte-specific knockdown of *Torso* shortened lifespan (Fig. 3*C*), which suggests that oenocytes might not be the direct target of PTTH for longevity control.

Since PG is the major larval tissue for ecdysone biosynthesis, our PG-specific *Torso* knockdown indicates that ecdysone signaling is involved in PTTH-regulated longevity. To test this idea, we fed wild-type or *Ptth* mutants with 20-hydroxyecdysone (20E) at the beginning of L3 larval stage and then monitored the adult lifespan. We have previously shown that feeding 20E to *Ptth* mutant larvae rescued the development delay (26). As expected, 20E feeding during larval development abolished the lifespan extension of *Ptth* mutants (Fig. 3*D*). Consistently, knockdown of ecdysone receptor (*EcR*) specifically in oenocytes prolonged the lifespan (Fig. 3*E*), and blunted age-dependent increases in nuclear translocation of Relish in oenocytes (Fig. 3*G*). Together, these data suggest that PTTH regulates lifespan through its receptor Torso and ecdysone signaling during *Drosophila* development.

### PTTH Regulates Metabolic and Developmental Processes during the Larva-to-Adult Transition.

**Fig. 4.**
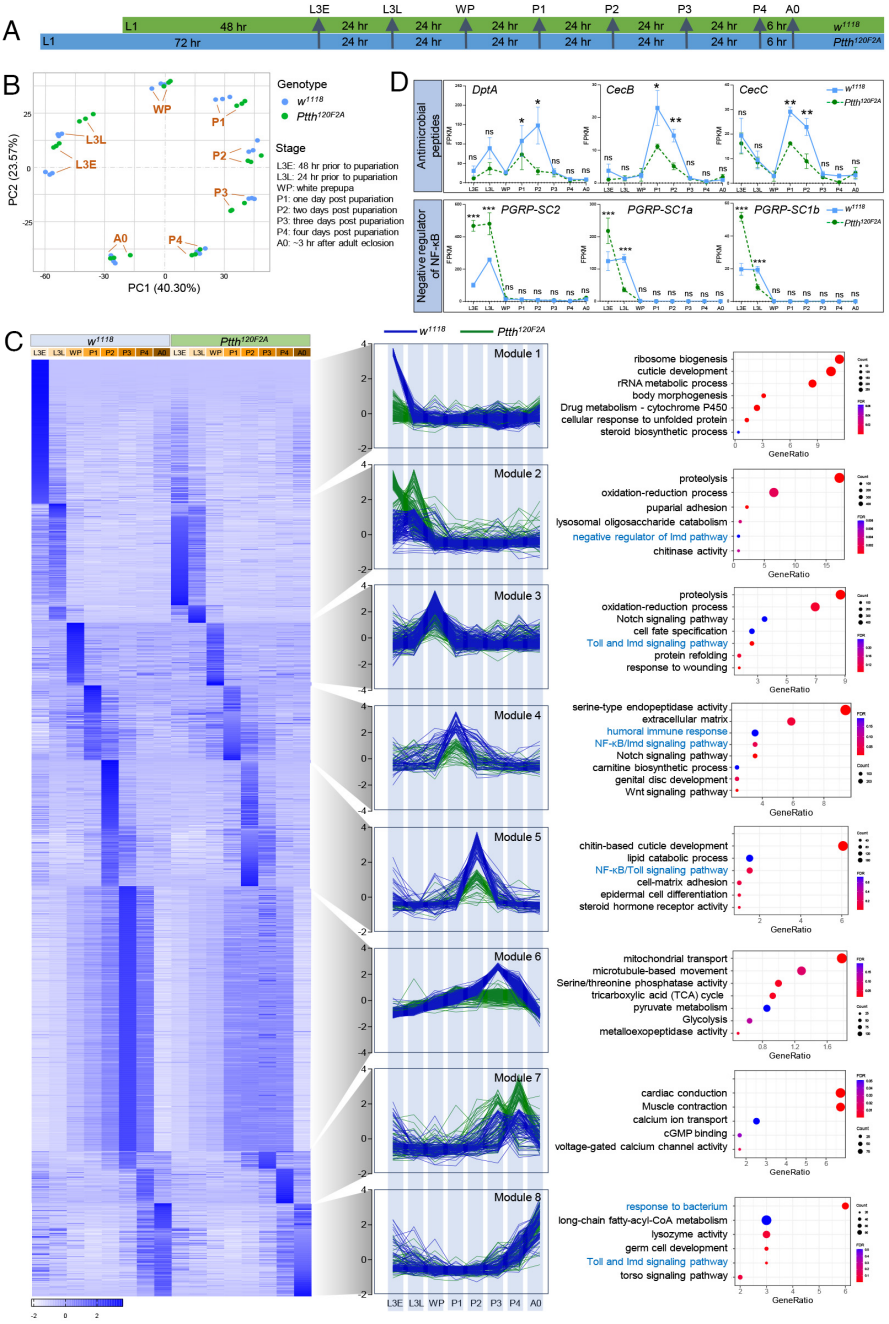
PTTH regulates metabolic and developmental processes, as well as NF-κB signaling during the larva-to-adult transition. (*A*) Schematic diagram showing the sample collection times for the development transcriptomic profiling. (*B*) PCA plot showing stage-specific transcriptomic profiling of wild-type (*w^1118^*) and *Ptth* mutants (*Ptth^120F2A^*). (*C*) Heat map, line plots, and pathways analysis for eight distinct clusters identified from 4,000 DEGs between wild-type (*w^1118^*) and *Ptth* mutants (*Ptth^120F2A^*) at different developmental stages. (*D*) Expression of antimicrobial peptide genes and negative regulation of NF-κB in wild-type (*w^1118^*) and *Ptth* mutants (*Ptth^120F2A^*) at different developmental stages. FPKM expression values are retrieved from the RNA-seq analysis. Two-way ANOVA followed by Bonferroni’s multiple comparison test. ns, not significant; *P < 0.05; **P < 0.01, n = 3.

In contrast to their role in initiating metamorphosis, the downstream target processes of PTTH-Torso-EcR signaling during *Drosophila* development are largely unknown. To unbiasedly identify developmental processes through which PTTH regulates lifespan, we performed a developmental RNA-Seq to profile the temporal dynamics of transcriptomic changes throughout larva-to-adult development in both wild-type and *Ptth* mutants. Eight developmental stages were used: L3E (early 3rd instar larvae, 48 h prior to pupariation), L3L (late 3rd instar larvae, 24 h prior to pupariation), WP (white prepupa), P1 (1 d post pupariation), P2 (2 d post pupariation), P3 (3 d post pupariation), P4 (4 d post pupariation), A0 (~3 h after adult eclosion) (Fig. 4*A*). Principal component analysis (PCA) revealed that the biological replicates for each developmental stage grouped together (Fig. 4*B*). The eight developmental groups, regardless of wild-type or *Ptth* mutants, were arranged nicely following the developmental trajectory from L3E to A0 (Fig. 4*B*). In addition, there was a clear separation between wild-type and *Ptth* mutants in the two 3rd instar larval stages (L3E and L3L), as well as the two early pupal stages (P1, P2) (Fig. 4*B*).

The volcano plot analysis showed more genes were differentially regulated by *Ptth* mutants at the 3rd instar larval stages (*SI Appendix*, Figs. S6 and S7*A*). In addition, the differentially regulated biological processes enriched for each developmental stage were also distinct. During larval development (L3E, L3L), cytoplasmic translation and ribosome biogenesis were differentially regulated by *Ptth* mutants. During pupal development, various metabolic and developmental processes were differentially regulated by *Ptth* mutants, such as cell differentiation, cell morphogenesis, epithelium development, and nervous system development (*SI Appendix*, Fig. S7*B*).

To further characterize the biological processes that were enriched for specific developmental stages and specific genotypes, we performed DESeq2 differential expression analyses (44) to identify stage-specific genes for each genotype respectively (fold change > 2, FDR < 0.05), followed by DEG identification by comparing wild-type samples and *Ptth* mutant samples at each developmental stage (see *Materials and Methods* section for more details). We identified a total of 4,313 DEGs that were differentially expressed between wild-type and *Ptth* mutants across eight different developmental stages (*SI Appendix*, Fig. S7*A*). The DEGs belonged to eight distinct coexpression gene modules (Fig. 4*C*). Each module represented a cluster of genes highly coexpressed at one or two specific developmental stages in wild-type samples. For example, Module 1 includes genes that were specifically induced at L3E stage in wild-type samples, whereas Module 3 includes genes that were specifically induced at WP stage in wild-type samples (Fig. 4*C*). Among the eight gene modules, loss of *Ptth* resulted in a decreased gene expression in most of the modules, except for Module 2 (L3L) and Module 7 (P3, P4) (Fig. 4*C*). Strong reduction of gene expression by *Ptth* mutants was observed in Module 1 (L3E) and Module 6 (P3). Module 1 included genes involved in ribosome biogenesis, cuticle development, rRNA metabolic process, steroid biosynthetic process, and body morphogenesis. The differentially regulated steroid biosynthetic process (such as ecdysteroids) by *Ptth* mutants is consistent with previous studies (26). Module 6 included genes in mitochondrial transport, microtubule-based movement, serine/threonine phosphatase activity, tricarboxylic acid (TCA) cycle, pyruvate metabolism, and glycolysis (Fig. 4*C*). Similar results were observed using multiWGCNA gene coexpression analysis (*SI Appendix*, Fig. S7*C*). Thus, PTTH mutants exhibit stage-specific transcriptomic changes, and alter many novel metabolic and developmental processes during larva-to-adult transition.

### NF-κB Signaling Is Downregulated in *Ptth* Mutants during *Drosophila* Development.

Intriguingly, our developmental RNA-Seq analysis revealed temporal dynamics of gene expression associated with innate immunity and NF-κB signaling pathways (Fig. 4*C*). The role of NF-κB signaling in *Drosophila* development and metamorphosis is largely unexplored. Recent studies show that hematopoietic niche-expressing Relish/NF-κB is required to maintain the blood progenitors in developing lymph glands (35), while retrotransposon activation during *Drosophila* pupal stage primes the host antiviral responses via the induction of NF-κB signaling (36). Through our developmental RNA-Seq analysis, we found that in wild-type animals, many AMP genes (e.g., *DptA, CecB, CecC, AttA, DptB, Mtk, Dro,*) showed peak expression at both 3rd instar larval (L3E, L3L) and early pupal stages (P1, P2) (Fig. 4*D* and *SI Appendix*, Fig. S5*B*), which corresponds to the rise of *Ptth* expression during larval development and metamorphosis (*SI Appendix*, Fig. S5*A*). In addition, there were other AMPs (e.g., *AttB, AttC, Drs*) that showed a peak expression only at 3rd instar larval stages (L3E, L3L) (*SI Appendix*, Fig. S5*B*), while the expression of two AMPs (*Def and Drsl2*) peaked only at early pupal stages (*SI Appendix*, Fig. S5*B*). These data indicate that NF-κB signaling is activated during the larva-to-adult transition, especially at 3rd instar larval and early pupal stages. Our findings also suggest that NF-κB signaling plays an important but underappreciated role in regulating *Drosophila* development and metamorphosis (juvenile-to-adult transition).

Strikingly, NF-κB signaling and innate immunity were differentially regulated by *Ptth* mutants in five out of eight coexpression modules (Module 2, 3, 4, 5, 8) (Fig. 4*C*). Almost all AMP genes were significantly downregulated by *Ptth* mutants at both 3rd instar larval and early pupal stages (Fig. 4*D* and *SI Appendix*, Fig. S5*B*). In addition, the expression of three negative regulators of NF-κB signaling (*PGRP-SC2*, *PGRP-SC1a*, *PGRP-SC1b*) peaked at 3rd instar larval stages (L3E, L3L) in wild-type, while loss of *Ptth* increased the expression of these negative regulators, in particular *PGRP-SC2* (Fig. 4*D*). Together, these data suggest a strong link between PTTH and NF-κB signaling during *Drosophila* development and metamorphosis, and PTTH might act as an upstream regulator that activates developmental NF-κB signaling during the juvenile-to-adult transition in *Drosophila*.

**Fig. 5.**
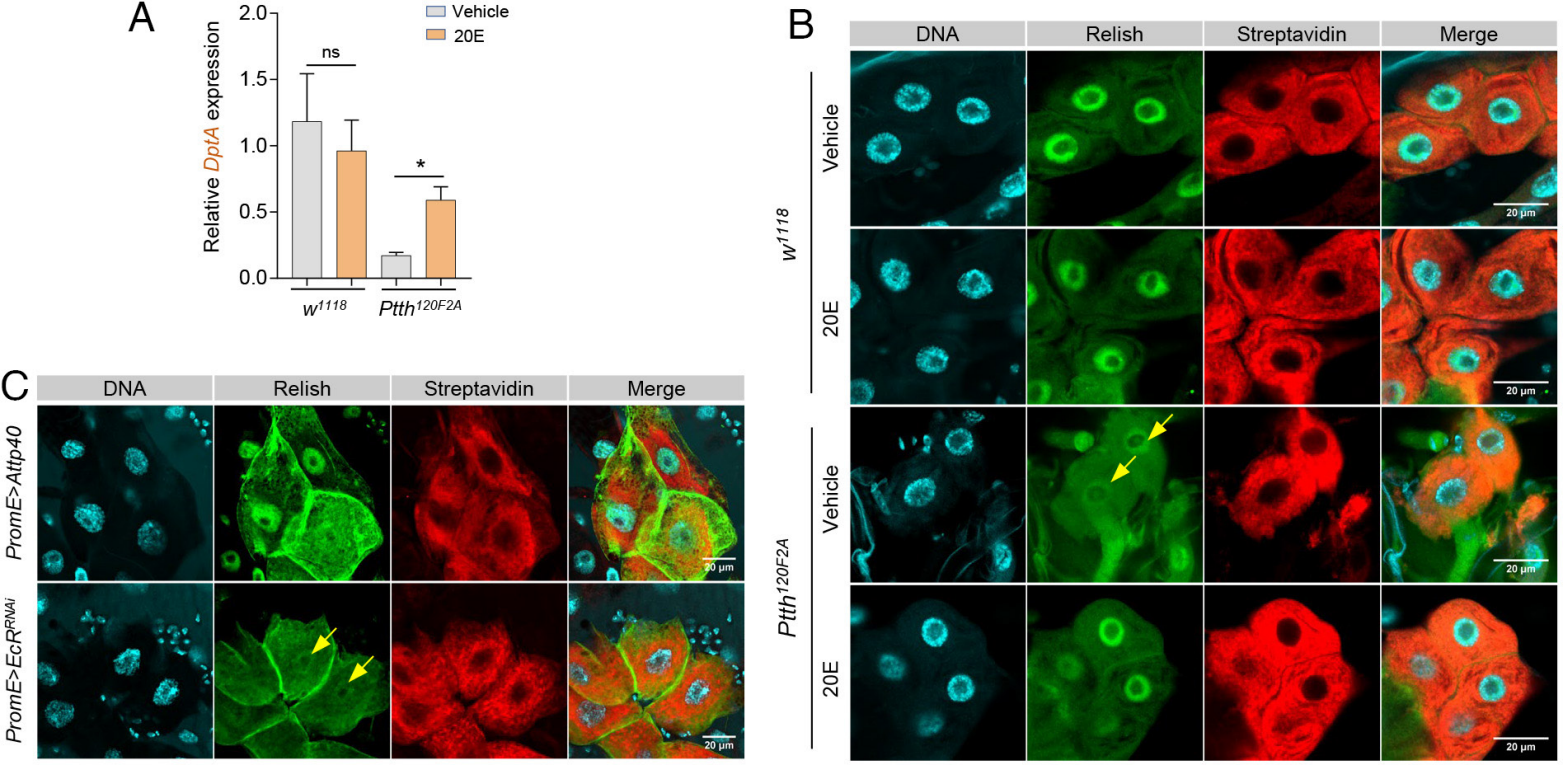
PTTH regulates developmental NF-κB signaling through ecdysone. (*A*) qPCR analysis of the expression of *DptA* in 3rd instar larvae of wild-type and *Ptth* mutants upon 20E feeding. Two-way ANOVA followed by Bonferroni’s multiple comparison test. ns, not significant; *P < 0.05, n = 6. (*B*) Immunostaining analysis of nuclear translocation of Relish/NF-κB in oenocytes of late 3rd instar larvae (L3L) in wild-type and *Ptth* mutants upon 20E feeding. Larval oenocytes were marked by streptavidin counterstaining. (Scale bar: 20 μm.) Yellow arrows: the nuclei with reduced Relish translocation. (*C*) Immunostaining analysis of nuclear translocation of Relish/NF-κB in oenocytes of late 3rd instar larvae (L3L) in oenocyte-specific *EcR* knockdown. Larval oenocytes were marked by streptavidin counterstaining. (Scale bar: 20 μm.) Yellow arrows: the nuclei with reduced Relish translocation.

One possibility for PTTH-regulated developmental NF-κB signaling is through ecdysone. To test this idea, we again fed wild-type or *Ptth* mutants with 20E at 1st instar larval stage and then measured NF-κB signaling at late 3rd instar larval stage (L3L). As expected, 20E feeding partially restored the low *DptA* transcription of *Ptth* mutants (Fig. 5*A*). We further showed that feeding 20E to *Ptth* mutants enhanced the nuclear translocation of Relish compared to vehicle control, specifically within larval oenocytes [indicated by streptavidin counterstaining (45)] (Fig. 5*B*). In addition, oenocyte-specific knockdown of *EcR* blocked the activation of NF-κB signaling, indicated by the nuclear translocation of Relish, in oenocytes of 3rd instar larvae (Fig. 5*C*). Together, our data demonstrate that PTTH induces NF-κB signaling through ecdysone in oenocytes during the juvenile-to-adult transition in *Drosophila*.

### Silencing *Relish/NF-κB* in Developing Hepatocytes Leads to Developmental Delay and Lifespan Extension.

**Fig. 6.**
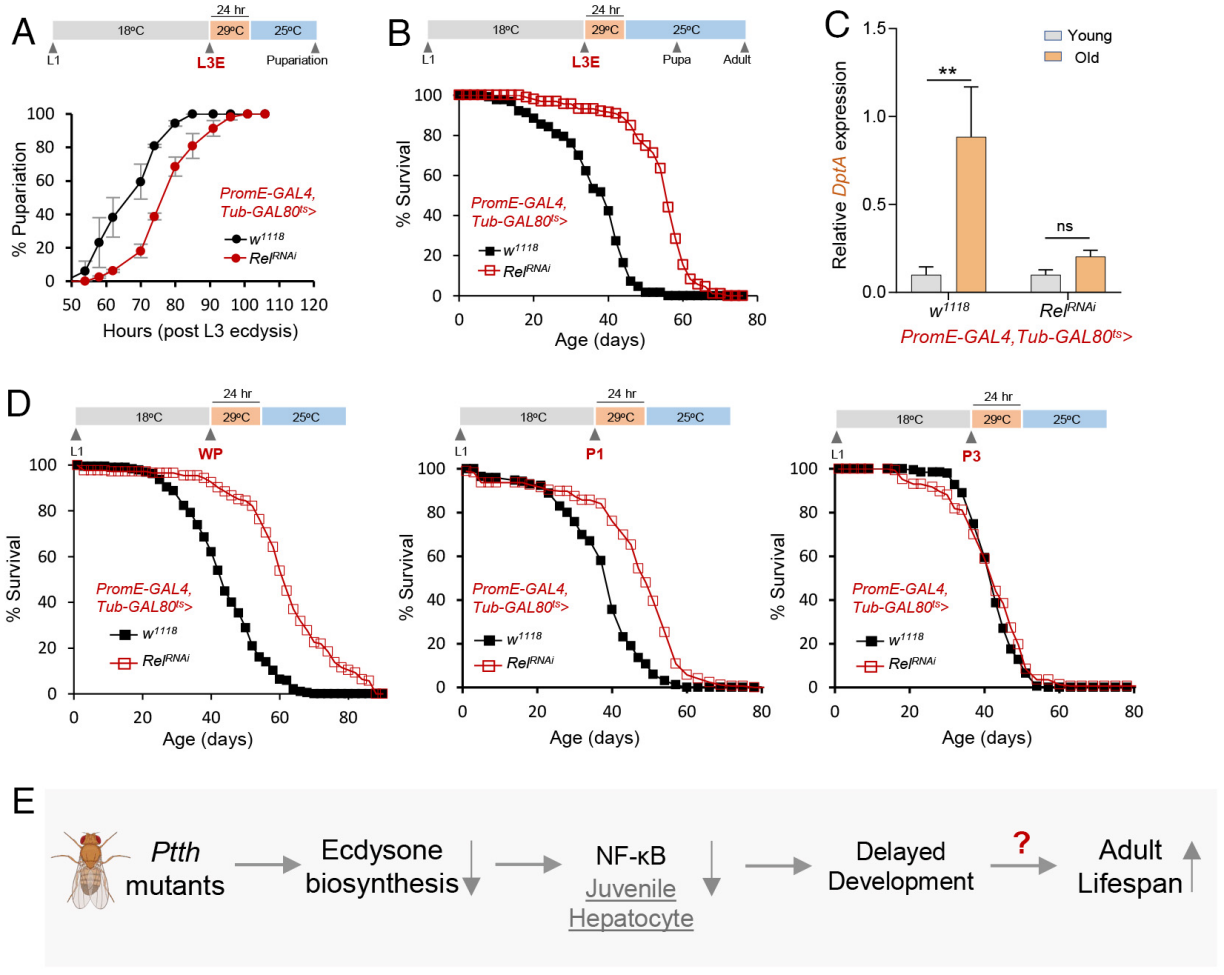
Silencing *Relish/NF-κB* in larval oenocytes delays development and prolongs lifespan. (*A*) Developmental timing analysis of oenocyte-specific knockdown of *Relish* at early 3rd instar larval stage (L3E). Schematic diagram of the experimental design is shown above the data. *PromE-GAL4; Tub-GAL80^ts^* was used to drive time-restricted gene silencing in larval oenocytes. Larvae were incubated at 29 °C for 24 h to activate RNAi. Two replicates were performed for each genotype (about 30 to 40 larvae each replicate). (*B*) Lifespan analysis of oenocyte-specific knockdown of *Relish* at early 3rd instar larval stage (L3E). Log-rank test, *P* < 0.001, n = 229. (*C*) qPCR analysis of the expression of *DptA* in young and old flies with short-term oenocyte-specific knockdown of *Relish* at early 3rd instar larval stage (L3E). Two-way ANOVA followed by Bonferroni’s multiple comparison test. ns, not significant; **P < 0.01, n = 6. (*D*) Lifespan analysis of oenocyte-specific knockdown of *Relish* at prepupa (WP) and different pupal stages (P1-P4). WP: Log-rank test, *P* < 0.001, n = 552. P1: Log-rank test, *P* < 0.001, n = 344. P2: Log-rank test, *P* < 0.001, n = 414. P3: Log-rank test, *P* > 0.1, n = 332. P4: Log-rank test, *P* < 0.01, n = 280. (*E*) The proposed model shows *Drosophila Ptth* mutants delay development and prolong lifespan through NF-κB signaling in larval oenocytes (juvenile hepatocytes).

Given that PTTH activates NF-κB signaling during *Drosophila* larva-to-adult transition, we next asked whether the activation of NF-κB signaling in developing oenocytes could be the underlying mechanism linking development time to adult lifespan observed in *Ptth* mutants. To test this idea, we used the temperature-sensitive GAL80 system (*GAL80^ts^*) to achieve temporal and spatial gene silencing during *Drosophila* development (Fig. 6*A*). Animals carrying *GAL80^ts^* and *GAL4* were maintained at 18 °C throughout larval development, and then switched to 29 °C at specific larval or pupal stages to activate *GAL4* expression and gene silencing for about 24 h before switching to 25 °C for either developmental timing or lifespan analysis. We first confirmed that the approach was efficient at silencing *Relish* during development using *Tub-GAL4; Tub-GAL80^ts^*. As shown in *SI Appendix*, Fig. S8 *A* and *B*, the activation of *Relish* RNAi at prepupa for 24 h led to a significant reduction of Relish mRNA expression at early pupal stages (1-d post knockdown), while *Relish* expression was restored back to wild-type levels in adulthood.

Using the GAL80 system, we first examined whether oenocyte-specific NF-κB signaling regulates *Drosophila* developmental timing. Importantly, we found that oenocyte-specific knockdown of *Relish* at early 3rd instar larval stage (L3E) led to a 12-h developmental delay (Fig. 6*A*). This finding suggests that the downregulation of NF-κB signaling in larval oenocytes is partially responsible for PTTH-regulated developmental delay. Notably, oenocyte-specific knockdown of *EcR* at early 3rd instar larval stage (L3E) also led to delayed development (*SI Appendix*, Fig. S8*C*). Intriguingly, oenocyte-specific knockdown of *Relish* at early 3rd instar larval stage (L3E) not only delayed development, but also prolonged adult lifespan (47% extension of median lifespan) (Fig. 6*B*). In addition, we noticed that silencing of NF-κB signaling in larval oenocytes had prolonged impacts on adult inflammaging. Flies developed from the larvae with oenocyte-specific *Relish* knockdown showed lower age-dependent induction of *DptA* transcription (Fig. 6*C*). Together, these findings uncover an underappreciated role of NF-κB signaling in animal developmental timing, and demonstrate that manipulation of developmental NF-κB signaling can significantly influence adult lifespan and inflammaging.

Given that the expression of AMP genes were downregulated by *Ptth* mutants at both 3rd instar larval and early pupal stages, we then asked whether silencing *Relish* during early pupal stages can also extend lifespan. Indeed, when *Relish* was knocked down in oenocytes at prepupal (WP) or early pupal stages (P1), but not late pupal stages (P3), the adult lifespan was greatly extended (Fig. 6*D*). We recognize that at late pupal stages (P3, P4), larval oenocytes are degenerated and adult oenocytes have not been fully developed yet. Therefore, the *PromE-GAL4* driver may not be able to activate RNAi at late pupal stages, which could confound our interpretation of late pupal knockdown results. Despite this limitation, our findings demonstrate that the specific stage of *Relish* knockdown is crucial to achieve lifespan extension effects. Last, we showed that the whole-body knockdown of *Relish* at prepupal stages (WP) did not prolong lifespan, rather it shortened adult lifespan (*SI Appendix*, Fig. S8*D*). This finding suggests that silencing NF-κB signaling in tissues other than oenocytes could be detrimental. Indeed, our whole-body *Relish* knockdown results align well with a recent study showing that pupal-specific knockdown of *Relish* increases the susceptibility of adult flies to viral infection (36). Together, our studies suggest that NF-κB signaling acts on specific developing tissues and at specific developmental stages to regulate animal developmental timing and lifespan.

## Discussion

Developmental time (time to maturity) and maximum lifespan show a strong positive correlation. However, the genetic mechanisms underlying this phenomenon are largely unknown. One challenge is to establish a genetic model system that specifically alters developmental timing, as most of the longevity genes identified previously are the regulators for growth rate, but not developmental timing. For example, perturbation of developmental signaling pathways, such as GH (3, 6), insulin/IGF (4, 5), and mTORC1 signaling (46–49), often causes retarded growth and longer lifespan. In the present study, we target the major regulator of developmental timing in *Drosophila* and show that loss of *Ptth* results in extended lifespan and delayed developmental timing, which can be rescued through ecdysone feeding. We further show that loss of *Ptth* prolongs lifespan by repressing age-dependent induction of NF-κB signaling and chronic inflammation in fly hepatocytes (oenocytes). Intriguingly, PTTH is required for the activation of NF-κB signaling during larva-to-adult transition. Time-restricted and tissue-specific silencing of *Relish* in oenocytes during larval development leads to delayed pupariation, as well as extended lifespan. Thus, our work establishes a genetic model for dissecting the mechanisms linking developmental time to lifespan regulation. Our findings provide insights into the unexplored function of developmental NF-κB signaling in aging and longevity control (Fig. 6*E*).

Although both growth rate and developmental time are correlated with maximum lifespan, developmental time and maximum lifespan show a much stronger positive correlation (1, 14). In fact, no correlation between growth rate and maximum lifespan is found in birds (1). Our lifespan analysis using *Drosophila Ptth* mutants suggests that an individual’s developmental time determines lifespan, independent of the growth rate. We believe that *Ptth* mutants are excellent genetic model systems that uncouple developmental time from growth rate, which offers a great advantage in understanding the genetic mechanisms underlying the role of developmental time in longevity control. Our findings further exemplify that developmental time could be a better indicator of maximum lifespan than body size.

Our studies reveal that loss of *Ptth* leads to a downregulation of NF-κB signaling during the larva-to-adult transition in *Drosophila*. Our data suggest that PTTH regulates NF-κB signaling through ecdysone biosynthesis, since feeding 20E to *Ptth* mutants restore NF-κB signaling to wild-type levels. This finding is consistent with the previous studies showing that ecdysone signaling functions to prime the target tissues for rapid immune activation upon infection (50). Our studies using oenocyte-specific *EcR* knockdown further support the notion that ecdysone through its receptor EcR can activate innate immune signaling in larval oenocytes, which is likely mediated through the transcriptional activation of PGRC-LC as reported previously (50). However, what really excites us is that oenocyte-specific knockdown of *Relish* at early 3rd instar larval stage leads to delayed pupariation, as well as extended lifespan. The developmental effects of NF-κB signaling are understudied. A recent study reports that Gypsy retrotransposon is activated during *Drosophila* metamorphosis, which leads to the induction of NF-κB in a STING-dependent manner (36). However, it is unclear how manipulation of NF-κB signaling at early 3rd instar larvae can alter animal developmental timing. NF-κB is known to interact with various nutrient-sensing pathways (e.g., insulin/IGF-1 and mTOR) (51, 52), and involve in lipid metabolism and mitochondrial biogenesis and respiration (53). One possibility is that the perturbation of NF-κB signaling might alter metabolic and nutritional signaling pathways during larval development, which consequentially prolongs animal developmental time. Although PTTH has no mammalian homolog, *Ptth* mutants phenotypically resemble genetic mutations in mammalian growth factors, such as GH and insulin/IGF-1 signaling, in terms of retarded growth and prolonged lifespan (4–6). GH is known to interact with steroids (e.g., estrogens) to regulate animal growth and development (54). Our work might enlighten future investigations to see whether a similar growth factor—steroid—innate immunity axis exists in linking developmental time to lifespan regulation in mammals.

Our developmental RNA-Seq analysis uncovers two major peaks of AMP gene expression, at 3rd instar larval and early pupal stages. While the expression of AMP at 3rd instar larval stage might be related to larval developmental time, it is possible that the activation of NF-κB at early pupal stages contributes to the remodeling of adult tissues during *Drosophila* metamorphosis. Dorsal (Dl), a REL domain-containing protein of the NF-κB family, was first identified as a regulator of dorsoventral pattern formation during *Drosophila* embryogenesis (55). NF-κB signaling has also been shown to be activated in the hematopoietic niche to maintain blood progenitors in the developing lymph gland of *Drosophila* larvae (35). During zebrafish development, NF-κB is activated in endothelial cells to drive the specification of hematopoietic stem and progenitor cells (56, 57). Further, NF-κB is required for TNF-α-mediated osteogenic differentiation from the human dental pulp stem cells, a type of mesenchymal stem cells (58). NF-κB activation also promotes the migration and proliferation of human mesenchymal stem cells in response to proinflammatory cytokines, such as TNF-α and interleukin-1β (59, 60). Thus, it is possible that NF-κB signaling is activated in response to proinflammatory signals (or ecdysone signaling) during *Drosophila* metamorphosis, which in turn governs the remodeling of adult tissues. One such tissue could be adult oenocytes, a major tissue strongly associated with inflammaging (28). Our preliminary studies did not find any obvious morphological differences of adult oenocytes dissected from wild-type and *Ptth* mutants (*SI Appendix*, Fig. S8 *E* and *F*). Future single-cell transcriptomics analysis could help reveal more details on adult tissue remodeling in *Ptth* mutants through stage- and cell type–specific gene expression analysis.

PTTH is well known for its role in controlling developmental timing through ecdysteroid biosynthesis (21, 26). However, whether PTTH also regulates other developmental processes beyond ecdysteroid biosynthesis is largely unknown. Our developmental RNA-Seq analysis reveals that PTTH targets many unexplored metabolic and developmental processes. These developmental processes include cell morphogenesis, epithelium development, nervous system development, and cuticle development. The metabolic processes include ribosome biogenesis, tricarboxylic acid (TCA) cycle, pyruvate metabolism, and glycolysis. These findings are aligned with the target tissue of PTTH, the fly hepatocytes (oenocytes). We speculate that PTTH may regulate animal developmental timing through metabolic programming in metabolic tissues like the juvenile liver. Further studies are needed to investigate the metabolic changes in metabolic tissues during animal development, and whether these metabolic changes are correlated with developmental timing and regulated by PTTH.

In summary, we established a genetic model to dissect the link between developmental time and lifespan. Our studies revealed that the insect hormone PTTH regulates lifespan through developmental NF-κB signaling in oenocytes during *Drosophila* larval development and metamorphosis. The *Ptth* mutants represent unique genetic model systems that uncouple developmental time from growth rate, which offers many advantages in future interrogation of the genetic mechanisms linking developmental time to longevity control.

## Materials and Methods

Details on the RNA-seq analysis, bioinformatics analysis, immunostaining and confocal microscopy, lifespan analysis, molecular and biochemical analysis, and behavior analysis, can be found in *SI Appendix*, *SI Materials and Methods*.

## Supplementary Material

Appendix 01 (PDF)

## Data Availability

RNA-Seq data have been deposited in NCBI-GEO (GSE271165 and GSE271166) (61, 62). All data needed to evaluate the conclusions in the paper are present in the paper and/or *SI Appendix*.
