# Supplementary material for: NF-κB-mediated developmental delay extends lifespan in Drosophila
Source: Proc Natl Acad Sci U S A. 2025 May 8;122(19):e2420811122. doi: 10.1073/pnas.2420811122 (PMC12088391; doi:10.1073/pnas.2420811122)
Supplement: Supplementary file 1 — Appendix 01 (PDF) [file pnas.2420811122.sapp.pdf]

Supporting Information for
NF-κB-mediated developmental delay extends lifespan in *Drosophila*

Ping Kang [a,1], Peiduo Liu[a], Yanhui Hu[c], Jinoh Kim[a], Ankur Kumar[a], Marlene K. Dorneich-Hayes[a], Wren Murzyn[a], Zenessa J. Anderson[a], Lexi N. Frank[a], Nicholas Kavlock[a], Elizabeth Hoffman[a], Chad C. Martin[a], Ting Miao[a,2], MaryJane Shimell[b], Jo Anne Powell-Coffman[a], Michael B. O'Connor[b], Norbert Perrimon[c,d], Hua Bai [a,1]

[a]Department of Genetics, Development, and Cell Biology, Iowa State University, Ames, IA, USA
[b]Department of Genetics, Cell Biology and Development, University of Minnesota, Minneapolis, MN, USA
[c]Department of Genetics, Harvard Medical School, Boston, MA, USA
[d]Howard Hughes Medical Institute, Boston, MA, USA

[1] Corresponding Author:
Hua Bai, hbai@iastate.edu
Ping Kang, pkang@iastate.edu

[2]Present address: Department of Genetics, Harvard Medical School, Boston, MA, USA

This file includes:

Supplementary Methods and Materials
Figures S1 to S8

Supplementary Methods and Materials

Fly husbandry and stocks

Flies were maintained at 25°C, 60% relative humidity and 12-hour light/dark cycles. Adults were reared on agar-based diet with 0.8% cornmeal, 10% sugar, and 2.5% yeast (5% yeast was used during the larval development of *w^{1118}* and *$Ptth^{120F2A}$*). Fly stocks used in the present study are: *$Ptth^{120F2A}$*, *$Ptth^{TI}$* (RRID:BDSC_84568), *Da-GS-GAL4* (a gift from Marc Tatar), *Phm-GAL4*, *PromE-GAL4* (or *Desat1-GAL4.E800*, RRID:BDSC_65405), *PromE-GS-GAL4* (a gift from Heinrich Jasper), *PromE-GAL4/Tub-$GAL80^{ts}$* (RRID:BDSC_65407), *Tub-GAL4/Tub-$GAL80^{ts}$*, *UAS-Relish RNAi* (RRID:BDSC_28943), *UAS-FLAG-Rel68* (RRID:BDSC_55777), *UAS-Ptth-RNAi* (VDRC #102043), *UAS-Torso-RNAi* (VDRC #36280), *UAS-EcR-RNAi* (RRID:BDSC_29374). The following genotypes were used as control in the knockdown or overexpression experiments: *w^{1118}*, *yw^R* (a gift from Marc Tatar), *y^1 v^1; P[CaryP]attP40* (BDSC # 36304). Gene knockdown using GeneSwitch driver was achieved by feeding flies on 200 µM RU486 (mifepristone, Cayman Chemical).

Developmental timing and body weight analysis

To synchronize development for timed experiments, parental flies were allowed to lay eggs for 3~4 hours on an apple juice agar plate coated with a thin layer of yeast paste. Twenty or twenty-four hours after egg laying, newly hatched L1 larvae were transferred to fly culture vials. Larvae were raised in groups of 30~40 to prevent crowding. The time of pupariation was scored every 4 hours till all larvae molt into pupae. Pupariation data from 3 replicates were compiled and plotted in Excel or Graphpad.

To measure body weight, one-week-old adult flies were collected after brief anesthesia with FlyNap (Carolina Biological Supply). 20 flies were pooled and weighed together over a weighing paper.

Demography and survival analysis

Flies were collected under brief CO_2 anesthesia and placed in food vials at a density of 25~30 females/males flies per vial, with a total of 150~300 flies for most conditions. Flies were transferred to fresh food every other day, and dead flies were scored and counted. Survival analysis was conducted with JMP statistical software. Data from replicate vials were combined. Survival distributions were compared by Log-rank test.

Rescue by 20E feeding

For 20E rescue experiments (both developmental timing and demography), newly ecdysed wild-type and *Ptth* mutant L3 larvae were washed with water and transferred to fly vials containing either 0.5 mg/ml of 20E or vehicle (95% ethanol).

Climbing Assay

Climbing ability was measured via a negative geotaxis assay performed by tapping flies to the bottom of an empty glass vial and counting flies that climbed at different positions of the vial. Ten seconds after tapping, the percentage of flies in each section of the vial (0~3 cm, 3~6 cm, 6~9 cm) were counted. The climbing ability index was calculated by weighing the number of flies according to their positions on the test vial.

Female fecundity analysis

Three-day-old mated female flies were maintained on food for 10 days at 5 females per vial and 3 vials per group. Flies were daily passed to new vials, and eggs were counted daily. The mean number of daily egg-laying was plotted.

Oxidative stress resistance assay

To assess oxidative stress resistance, 5-day-old flies were transferred into glass vials containing 1% agar, 5% sugar, and 20 mM paraquat (Sigma, St. Louis, MO, USA). Dead flies were scored and counted every 4 hours. A total of 40~50 flies were used for each genotype (10 flies per vial). Survival differences were analyzed by the Log-rank test.

Bacterial challenge assay

Gram-negative pathogenic bacterium *Erwinia carotovora carotovora* 15 (*Ecc15*) was cultured overnight to obtain OD_{600} = 200. To assess the survival upon bacterial challenge, 5-day-old flies were infected with 1:1 mixture of 5% sucrose and 100X concentrated *Ecc15* overnight culture. The infection solution was added onto a filter disk that was placed over fly vials with 1% agar base. Dead flies were scored and counted every 4 hours. A total of 60~80 flies were used for each genotype (10 flies per vial). Survival differences were analyzed by the Log-rank test.

Food Intake assay

One-week-old flies were first starved for 2 hours and then transferred to fresh food containing 2.5% blue food dye (FD & C Blue Dye no. 1) (10 females per replicate). Two hours after feeding, flies were homogenized in 200 μl of distilled water. After 5 min of centrifugation at 12,000 xg, 100 μl of supernatants were transferred to a 96-well plate and the absorbance was measured at 629 nm using BioTek spectrophotometer (Agilent Technologies).

Quantification of L-amino acid, citrate, and malate

About 25 mg of one-week-old flies were homogenized in 250 µl of distilled water. After 15 min of centrifugation at 10,000 xg, 20 µl of supernatants were transferred to a 96-well plate containing reaction mix from L-Amino Acid Assay Kit, or Citrate Assay Kit, or Malate Assay Kit (Sigma). The absorbance was measured at 570 nm (L-amino acid and citrate) or 565 nm (malate) using BioTek spectrophotometer (Agilent Technologies).

Locomotor activity assay

One-week-old flies were individually transferred to glass behavior tubes containing fresh fly food at one end (12 behavior tube per genotype). Locomotor activity was measured as the count of movements across Infrared beams per one-minute bin using DAM5H Drosophila Activity Monitor (Trikinetics Inc.).

RNA extraction and Quantitative RT-PCR

Adult tissues (fat body, oenocyte, gut) were dissected in 1 × PBS before RNA extraction. For oenocyte dissection, we first removed the fat body through liposuction and then detached oenocytes from the cuticle using a small glass needle. Tissue lysis, RNA extraction, and cDNA synthesis were performed using Cells-to-CT Kit (Thermo Fisher Scientific). For whole-body RNA extraction, flies were collected on CO2 and transferred to a 1.7 ml centrifuge tube with a stainless-steel ball and 500 µl Trizol reagent (Thermo Fisher Scientific, Waltham, MA, USA) and homogenized with Tissuelyzer. About 15 flies were used per replicate. DNase-treated total RNA was quantified by Nanodrop, and about 500 ng of total RNA was reverse transcribed to cDNA using iScript cDNA Synthesis Kit (Bio-Rad, Hercules, CA, USA).

QRT-PCR was performed with a Quantstudio 3 Real-Time PCR System and PowerUp SYBR Green Master Mix (Thermo Fisher Scientific). Two to three independent biological replicates were performed with two technical replicates. The mRNA abundance of each candidate gene was normalized to the expression of *RpL32* for fly samples by the comparative CT methods. Primer sequences are listed in the following: RpL32: forward 5’-AAGAAGCGCACCAAGCACTTCATC-3’ and reverse 5’-TCTGTTGTCGATACCCTTGGGCTT-3’. PGRP-LC: forward 5’-TTTAACCTTCCTGCTGGGTATC-3’ and reverse 5’-TTGTCTGTAATCGTCGTCATCTC-3’. DptA: forward 5’-TTGCCGTCGCCTTACTTT-3’ and reverse 5’-CCTGAAGATTGAGTGGGTACTG-3’. upd3: 5’- forward 5’-TCTGGAAGCTTCTTTCCGGC-3’ and reverse 5’-GCGGTCAGCTGTCGTCATTT-3’. Socs36E: forward 5’-ACTACGGTTTAGCCAAATTGC-3’ and reverse 5’-TGGACCTCCGATTGTTTTCTCT-3’.

Immunostaining and imaging

To examine the nuclear translocation of Relish, adult oenocytes were dissected from one-day-old pupas or female flies in 1X PBS and then fixed in 4% paraformaldehyde for 15 min at room temperature. Tissues were washed with 1x PBS with 0.3% Triton X-100 (PBST) three times (~5 min each time), and blocked in PBST with 5% normal goat serum for 30 min. Tissues were then incubated overnight at 4 °C with anti-Relish primary antibodies (RayBiotech, #RB-14-0004,1:500) diluted in PBST, followed by the incubation with secondary antibodies obtained from Jackson Immuno Research for 1 hr at room temperature the next day. After three washes, tissues were mounted using ProLong Gold antifade reagent (Thermo Fisher Scientific) and imaged with an FV3000 Confocal Laser Scanning Microscope (Olympus). DAPI or Hoechst 33342 was used for nuclear staining. Larval oenocytes were marked with streptavidin Alexa Fluor 555 (Thermo Fisher Scientific).

Western blot analysis

About 30 µg of protein lysates were denatured with Laemmli sample buffer (Bio-Rad, Cat# 161-0737) at 95 ^{0}C for 5 minutes. Then proteins were separated by Mini-PROTEAN® TGX Precast Gels (Bio-Rad). Following incubation with primary and secondary antibodies, the blots were visualized with West Pico PLUS Chemiluminescent Substrate (Thermo Scientific). The following antibodies were used: anti-Sting (a gift from Alan Goodman, 1:2000), anti-β-actin (Cell Signaling Technology, #4967, 1:2000), Peroxidase Donkey Anti-Rabbit IgG (Jackson ImmunoResearch, # 711-035-152, 1:5000).

RNA-Seq and bioinformatics

Two bulk RNA-Seq analyses were performed separately to profile transcriptomic changes in two adult ages and eight developmental stages (see main text). Total RNA was collected from 10~15 larvae, pupal, or adult flies (three biological replicates in each condition) using the Trizol method (described above), followed by DNase treatment (Ambion). RNA concentration was quantified by Qubit RNA BR Assay Kit (Thermo Fisher Scientific). RNA-Seq libraries were constructed using either NEBNext Ultra Directional RNA Library Prep Kit for Illumina (New England Biolabs) or by Novogene RNA-Seq service. Poly(A) mRNA was isolated using NEBNext Oligo d(T)25 beads and fragmented into 200 nt in size. After first-strand and second-strand cDNA synthesis, each cDNA library was ligated with a NEBNext adaptor and barcoded with an adaptor-specific index. Libraries were pooled in equal concentrations and sequenced using Illumina HiSeq 3000 (at ISU DNA facility) and Novoseq 6000 platforms (at Novogene Inc.).

The RNA-Seq data processing was performed on Ubuntu system. FastQC was first performed to check the sequencing read quality and Fastx is used to filter the bad quality read from fastq. Then the raw reads were mapped to the *D. melanogaster* genome (Drosophila_melanogaster.BDGP6.22.98.chr.gtf) using

Star (https://github.com/alexdobin/STAR.git). Htseq-count was used to count the number of mapped reads on each gene and DE-seq2 (R package) was used to generate normalized data. After normalization, differentially expressed protein-coding transcripts were obtained using the following cut-off values: false discovery rate (FDR) ≤ 0.05 and fold change ≥ 1.5 or 2. RNA-Seq read files have been deposited to NCBI 's Gene Expression Omnibus (GEO) (Accession # GSE271165 and #GSE271166). To review GEO accession GSE271165: Go to https://www.ncbi.nlm.nih.gov/geo/query/acc.cgi?acc=GSE271165. Enter token ynsdowwonhsnvcn into the box. To review GEO accession GSE271166: Go to https://www.ncbi.nlm.nih.gov/geo/query/acc.cgi?acc=GSE271166. Enter token crqrggyijrkljan into the box.

To identify stage- and genotype-specific differentially expressed genes from the developmental RNA-Seq, the count matrix was prefiltered to remove low-expressing genes in which the maximum expression level of a sample across all time points was less than 3 FPKM. Prefiltering also removes rows that have a total count that is less than 6. The filtered count matrix was then smoothed and normalized by the default method of DESeq2 (vs1.42). Differential expression analyses for stage-specific genes were performed using DESeq2 (one-tailed Wald test) between any one stage over the other seven stages for each genotype respectively. Differentially expressed genes (DEGs) were selected per timepoint as the genes having absolute log2 fold change (log2FC) larger than 1 and adjusted P value less than 0.05. To evaluate the difference between *Ptth* mutants and wild-type in each stage, we performed differential expression analysis (DESeq2, two-tailed Wald test) using filtered and smoothed counts matrices. In addition, multiWGCNA analysis was conducted to identify differentially expressed gene modules using a minimum module size of 50, maximum module size of 1000 and a soft threshold power of 12. All modules were tested for stage specificity (PERMANOVA $p<10^{-4}$) given the genotype. Module genes are selected by choosing overlapping genes between corresponding wild-type and mutant modules along with visual inspection on heatmap.

Statistical analysis

GraphPad Prism 7 (GraphPad Software, La Jolla, CA) was used for statistical analysis. To compare the mean value of treatment groups versus that of control, unpaired t-test or one-way ANOVA (followed by Tukey's multiple comparison) was performed. The effects of genotype on various traits were analyzed by two-way ANOVA followed by Bonferroni's multiple comparison test. Data are represented as mean ± standard error in each figure.

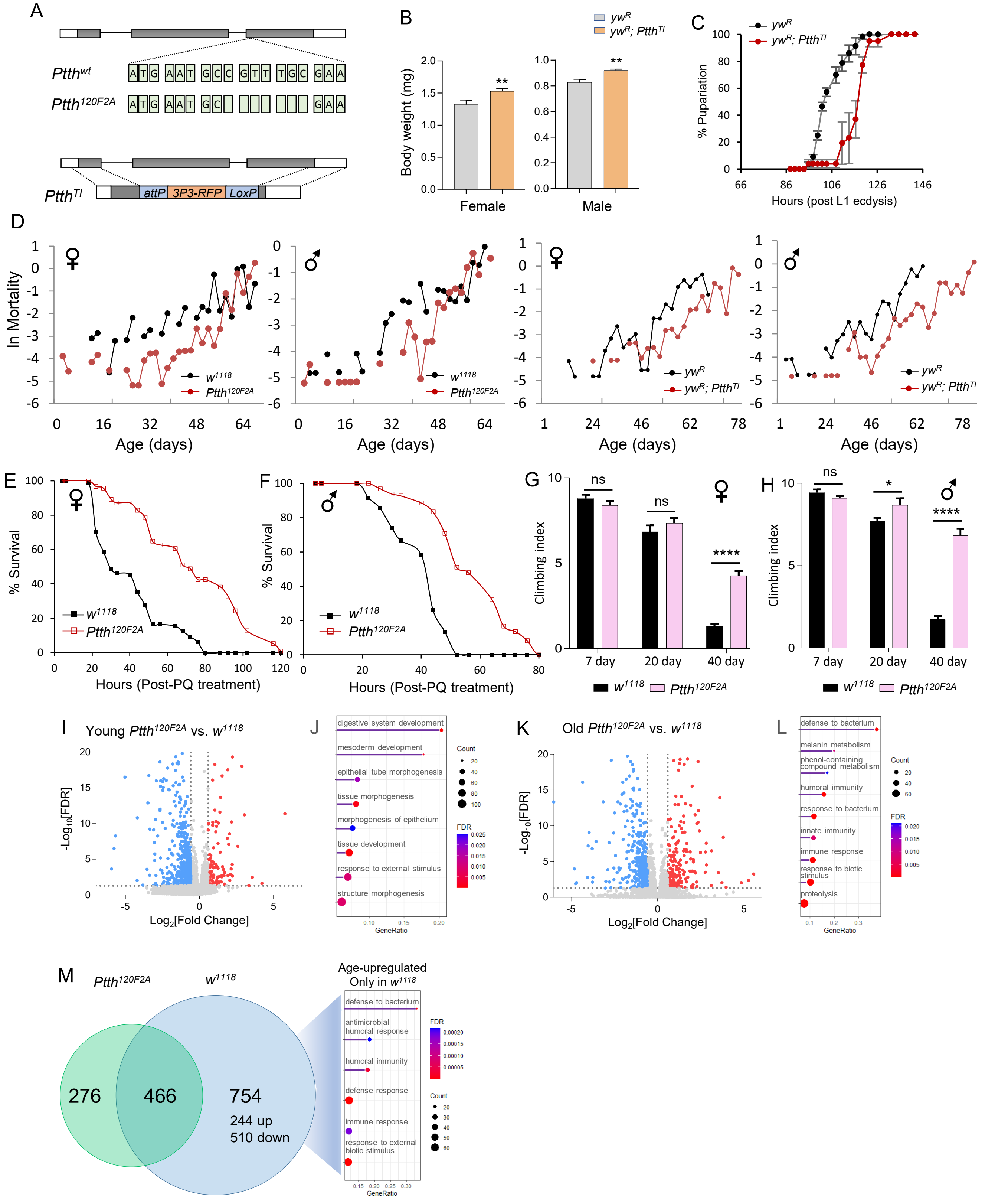

Fig S1. (*A*) Schematic diagram of two loss-of-function alleles of *Ptth*. (*B*) Body weight of 1-week-old adult wild-type (yw^R) and *Ptth* mutants ($Ptth^{TI}$). Unpaired t-test, ** $p < 0.01$. n = 4 (20 adults per replicate). (*C*) Developmental timing of wild-type (yw^R) and *Ptth* mutants ($Ptth^{TI}$). Three replicates were performed for each genotype (about 30~40 larvae each replicate). (D) Mortality rate plots of wild-type and *Ptth* mutants. Mortality rate, $\ln(\mu_x)$, is calculated as $\ln(-\ln(1-q_x))$, where q_x is age-specific mortality. (*E* and *F*) Survival analysis of *Ptth* mutants under 20 mM paraquat (PQ) treatment in both males and females. (log-rank test (vs. w^{1118}), $p < 0.001$, n = 100). (*G* and *H*) Climbing activity of female and male *Ptth* mutants during aging. Two-way ANOVA followed by Bonferroni's multiple comparison test. ns, not significant; * $p < 0.05$; **** $p < 0.0001$. n = 30. (*I* and *K*) Volcano plot showing genes significantly upregulated and downregulated by *Ptth* mutants at young (5-day-old) and old age (38-day-old). Fold change > 1.5, FDR < 0.05. (*J* and *L*) Dot plot analysis showing differentially regulated biological processes between *Ptth* mutants and wild-type (w^{1118}) at young and old ages. (*M*) Venn diagram and dot plot showing the number of age-upregulated genes in *Ptth* mutants and wild-type (w^{1118}), and the upregulated biological processes only found in wild-type (w^{1118}).

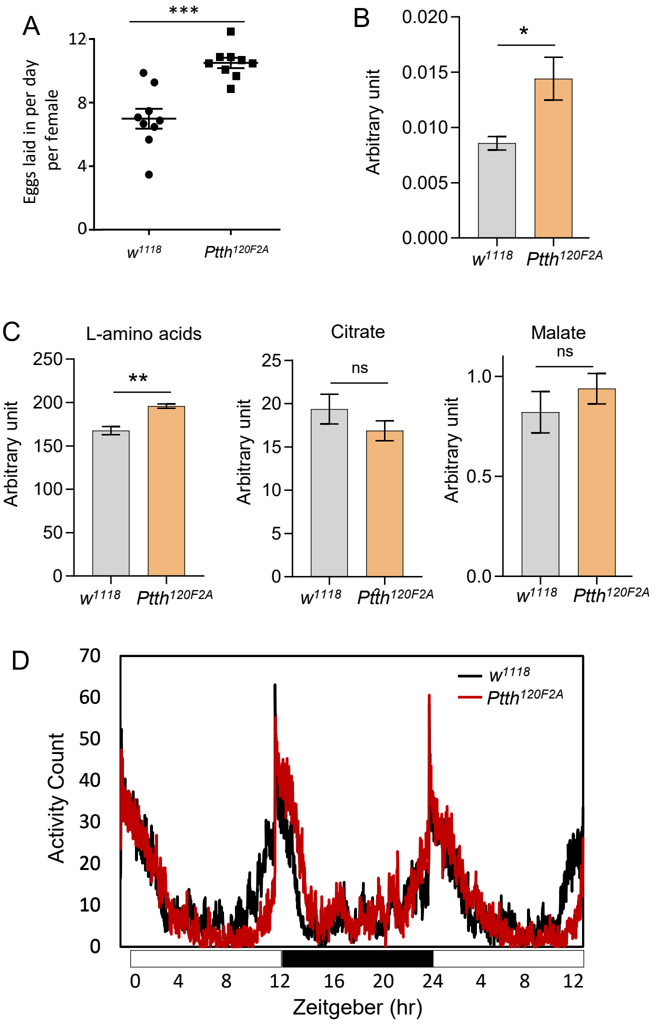

Fig S2. (*A*) Female fecundity analysis of 1-week-old wild-type and *Ptth* mutants. Unpaired t-test, *** $p < 0.001$. n = 9. (*B*) Food intake analysis of 1-week-old wild-type and *Ptth* mutants (female). Unpaired t-test, * $p < 0.05$. n = 6. (*C*) Quantification of L-amino acids, citrate, and malate in 1-week-old wild-type and *Ptth* mutants (female). Unpaired t-test, ** $p < 0.01$; ns, not significant. n = 3. (*D*) Actogram showing locomotor activity of 1-week-old wild-type and *Ptth* mutants (male).

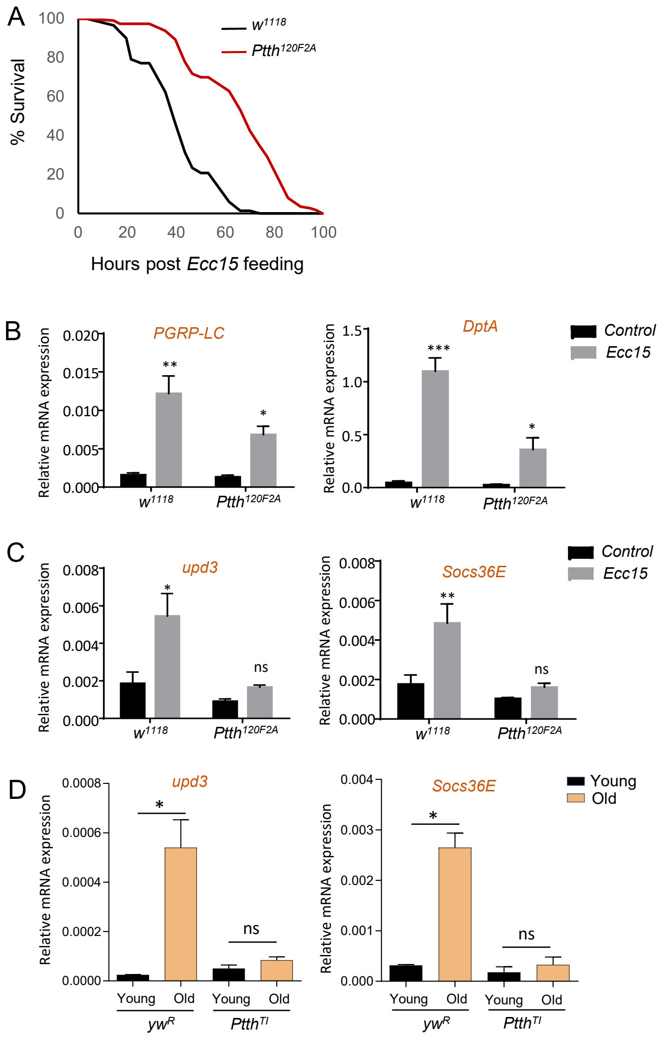

Fig S3. (*A*) Survival analysis of young wild-type and *Ptth* mutants upon *Ecc15* treatment (female). Log-rank test, $p < 0.001$, n = 125. No mortality found in 5% sucrose control group. (*B* and *C*) The expression of *PGRP-LC, DptA, upd3*, and *Socs36E* of young wild-type and *Ptth* mutants upon 16 hours of *Ecc15* treatment (female). One-way ANOVA followed by Tukey's multiple comparison test. ns, not significant; * $p < 0.05$; ** $p < 0.01$; *** $p < 0.001$. n = 3. (*D*) The expression of *upd3*, and *Socs36E* of young and old wild-type and *Ptth* mutants (female). One-way ANOVA followed by Tukey's multiple comparison test. ns, not significant; * $p < 0.05$. n = 3.

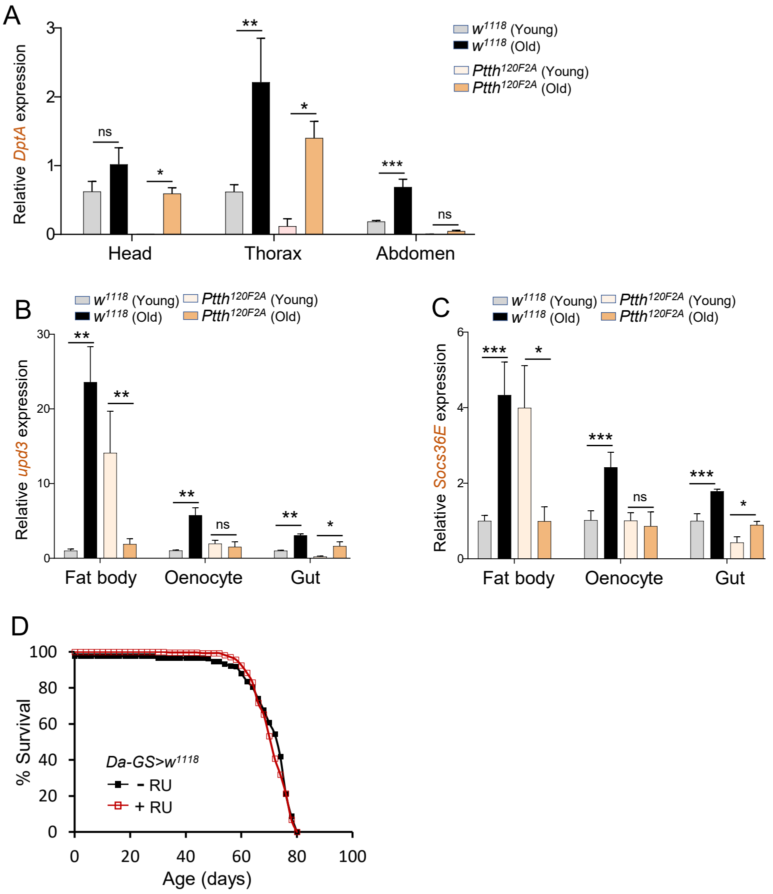

Fig S4. (*A*) qPCR analysis of the expression of *DptA* in three body parts dissected from young and old *Ptth* mutants and wild-type (w^{1118}). Two-way ANOVA followed by Bonferroni's multiple comparison test. ns, not significant; * $p < 0.05$; ** $p < 0.01$; *** $p < 0.001$. n = 4. (*B* and *C*) qPCR analysis of the expression of *upd3* and *Socs36E* in three different fly tissues dissected from young and old *Ptth* mutants and wild-type (w^{1118}). Two-way ANOVA followed by Bonferroni's multiple comparison test. ns, not significant; * $p < 0.05$; ** $p < 0.01$; *** $p < 0.001$. n = 3~6. (*D*) Lifespan analysis to test the effects of RU486 on lifespan. Log-rank test, $p > 0.05$, n = 485.

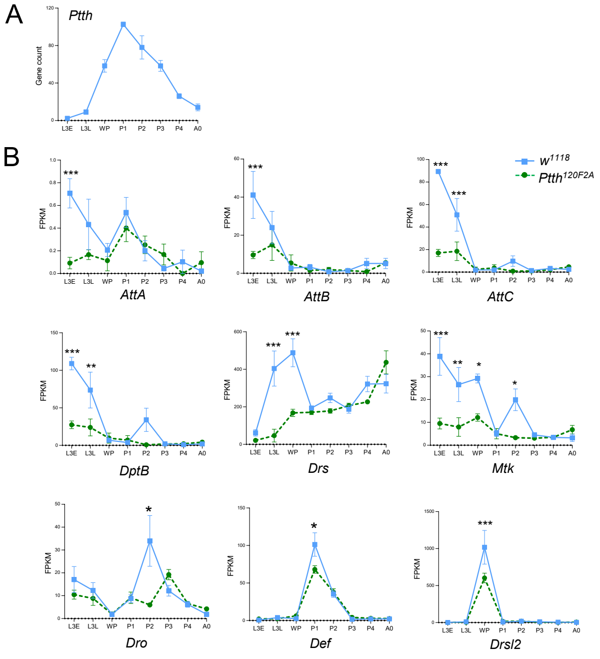

Fig S5. (*A*-*B*) Expression of *Ptth* and antimicrobial peptide genes and negative regulation of NF-κB in wild-type (w^{1118}) and *Ptth* mutants ($Ptth^{120F2A}$) at different developmental stages. FPKM expression values are retrieved from the RNA-seq analysis. Two-way ANOVA followed by Bonferroni's multiple comparison test. * $p < 0.05$; ** $p < 0.01$. n = 3.

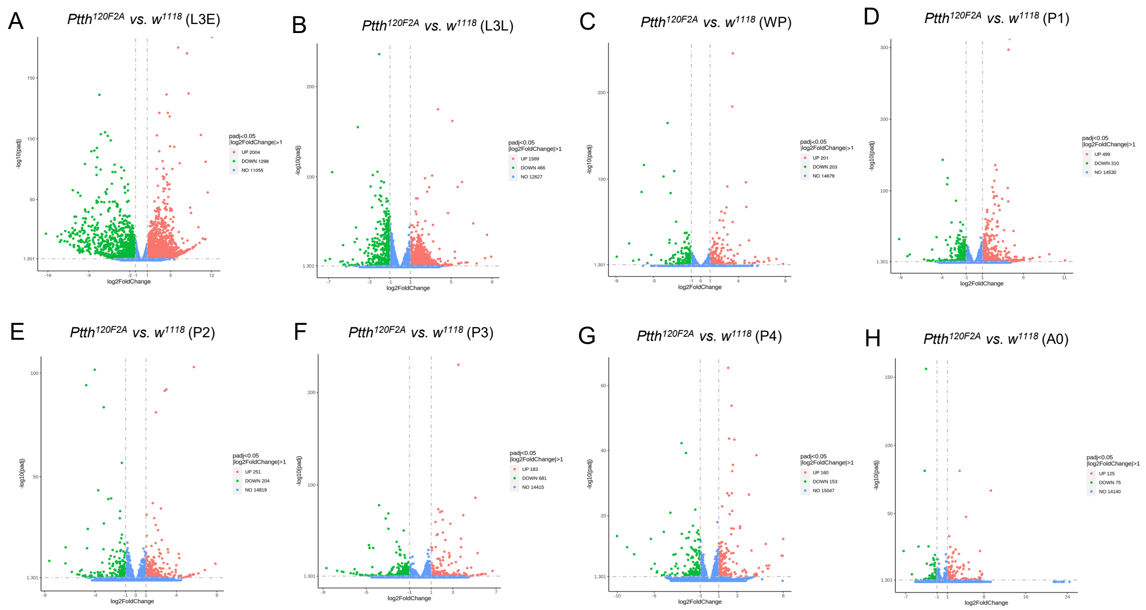

Fig S6. (*A-H*) Volcano plot analysis showing genes significantly upregulated and downregulated by *Ptth* mutants at different developmental stages. Fold change > 1.5, FDR < 0.05.

A

Stage	DEGs (w^{1118} vs. $Ptth^{120F2A}$)	up genes	down genes	Criteria
L3E	3049	1812	1237	fc>2, FDR<0.05
L3L	1896	1458	438	fc>2, FDR<0.05
WP	369	165	204	fc>2, FDR<0.05
P1	633	384	249	fc>2, FDR<0.05
P2	356	203	153	fc>2, FDR<0.05
P3	580	147	433	fc>2, FDR<0.05
P4	262	138	124	fc>2, FDR<0.05
A0	197	124	73	fc>2, FDR<0.05
Note: Total non-overlapping genes are 4313				

B

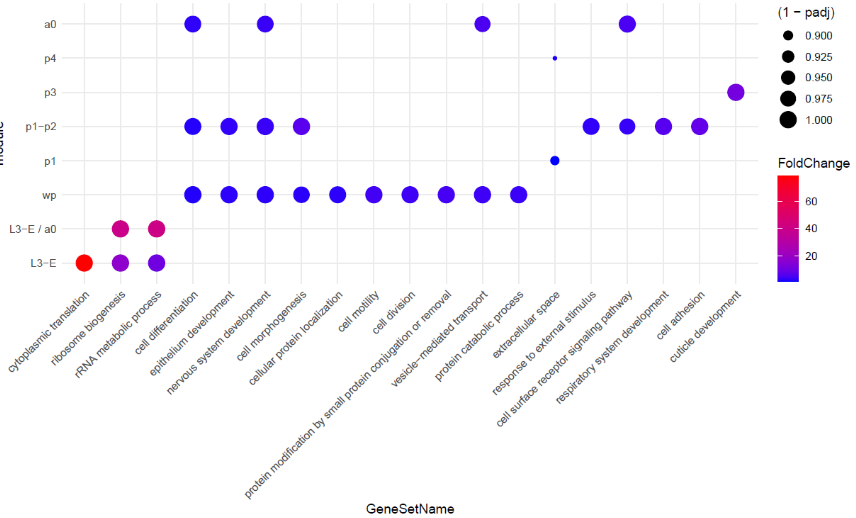

C

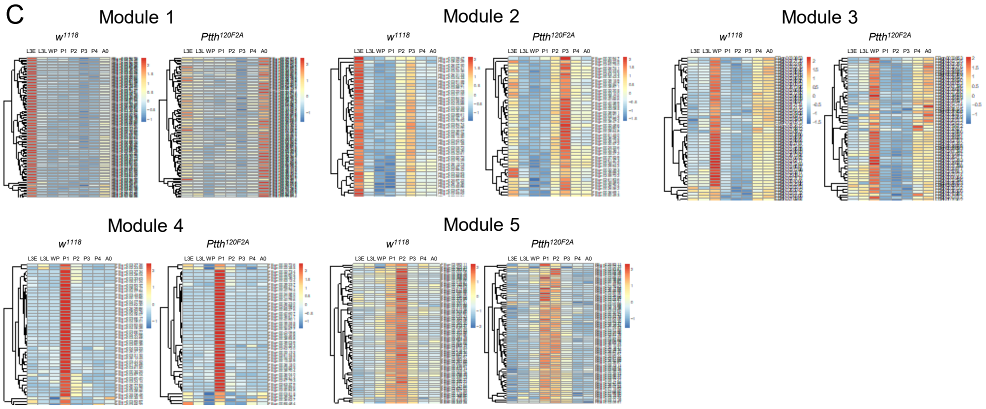

Fig S7. (*A*). The number of differentially expressed genes between wild-type and *Ptth* mutants across eight different developmental stages. (*B*) GO term analysis for the biological processes enriched in different developmental stages. (*C*) Five distinct modules identified by WGCNA analysis to show differentially expressed genes in wild-type (w^{1118}) and *Ptth* mutants at different developmental stages.

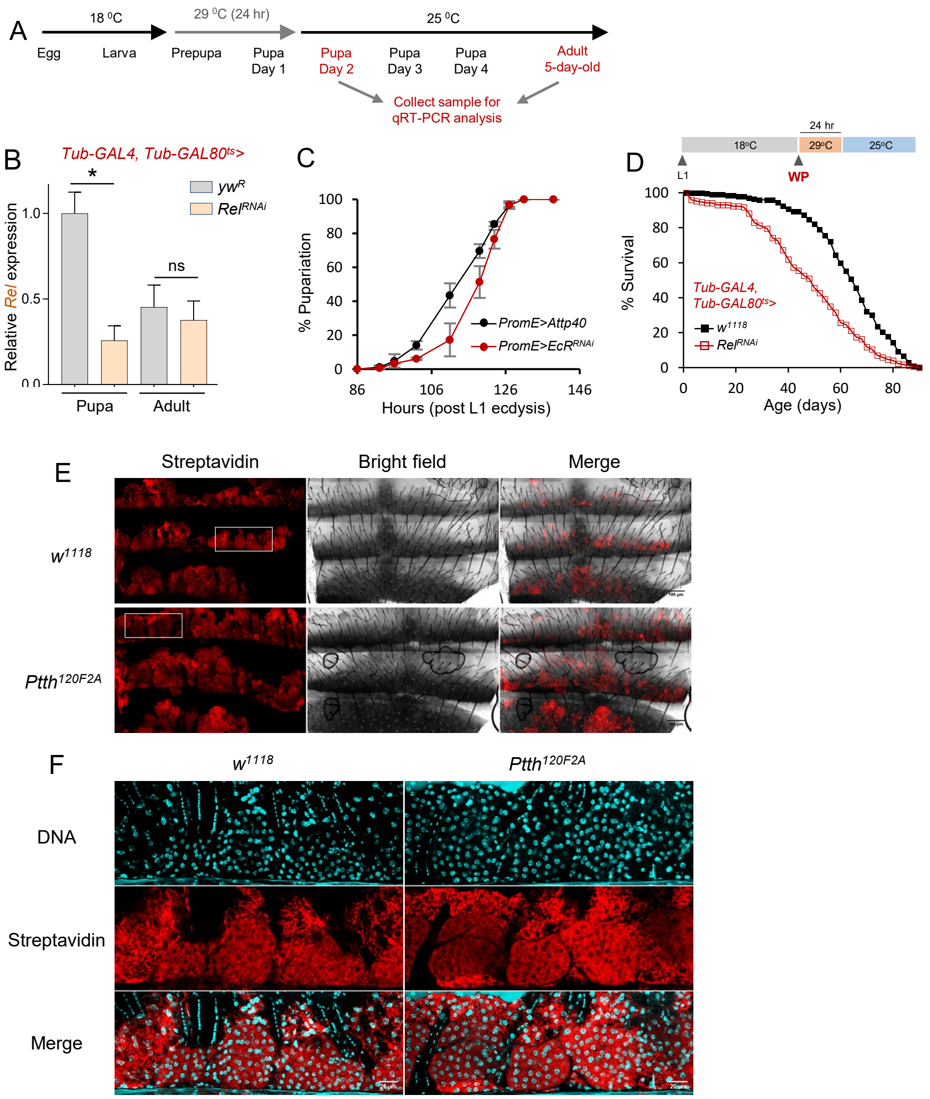

Fig S8. (*A*) Schematic diagram showing the design of sample collection post *Relish* knockdown. *Relish* RNAi was activated at prepupal stage. The samples at two following time-points are collected, one day or eight days after knockdown. (*B*) qRT-PCR analysis of the expression of *Relish* post knockdown at prepupal stage. One-way ANOVA followed by Tukey's multiple comparison test. ns, not significant; * p < 0.05. n = 3. (*C*) Developmental timing analysis of oenocyte-specific knockdown of *EcR*. Two replicates were performed for each genotype (about 30~40 larvae each replicate). (*D*) Lifespan analysis of whole body-knockdown of *Relish* at prepupal stage (WP). Log-rank test, p < 0.001, n = 569. (*E*) Immunostaining analysis of adult oenocyte morphology in wild-type and *Ptth* mutant flies (1-week-old). Oenocytes were marked by streptavidin counterstaining. Scale bar: 100 μm. White box: the area shown in Panel F. (*F*) Immunostaining analysis of adult oenocyte morphology in wild-type and *Ptth* mutant flies (1-week-old). Zoom-in images from Panel E.
